# High‐Throughput Kinetic Analysis for Target‐Directed Covalent Ligand Discovery

**DOI:** 10.1002/anie.201711825

**Published:** 2018-03-26

**Authors:** Gregory B. Craven, Dominic P. Affron, Charlotte E. Allen, Stefan Matthies, Joe G. Greener, Rhodri M. L. Morgan, Edward W. Tate, Alan Armstrong, David J. Mann

**Affiliations:** ^1^ Department of Chemistry Imperial College London South Kensington Campus London SW7 2AZ UK; ^2^ Department of Life Sciences Imperial College London South Kensington Campus London SW7 2AZ UK

**Keywords:** Cdk2, covalent inhibition, fragment-based drug discovery, kinetics, protein modification

## Abstract

Cysteine‐reactive small molecules are used as chemical probes of biological systems and as medicines. Identifying high‐quality covalent ligands requires comprehensive kinetic analysis to distinguish selective binders from pan‐reactive compounds. Quantitative irreversible tethering (qIT), a general method for screening cysteine‐reactive small molecules based upon the maximization of kinetic selectivity, is described. This method was applied prospectively to discover covalent fragments that target the clinically important cell cycle regulator Cdk2. Crystal structures of the inhibitor complexes validate the approach and guide further optimization. The power of this technique is highlighted by the identification of a Cdk2‐selective allosteric (type IV) kinase inhibitor whose novel mode‐of‐action could be exploited therapeutically.

Small molecules that bind proteins selectively serve both as tools to understand protein function and as therapeutics. Although high‐throughput screening has generated ligands against many clinical targets, a large subset appears undruggable as they lack deep hydrophobic pockets.^1^ Fragment‐based ligand discovery (FBLD) is a complementary technique that is able to generate ligands against many difficult‐to‐target proteins.^2^ Early strategies focused on non‐covalent fragments; however, recently FBLD has begun to incorporate electrophilic molecules.^3^, ^4^, ^5^, ^6^, ^7^, ^8^, ^9^, ^10^, ^11^ These fragments form covalent bonds with nucleophilic amino acids on target proteins and consist of a specificity‐determining element and a reactive warhead. Irreversible covalent modification proceeds under kinetic control and when developing irreversible inhibitors, warhead reactivity must be minimized and only become significant upon target‐engagement by the specificity element. Acrylamides, which modify cysteine residues, have recently become the most clinically successful covalent warhead.^12^

Screening irreversible cysteine‐reactive molecules by comparing their rates of reaction with a target protein alone is highly problematic as intrinsic electrophilic reactivity can vary dramatically, even for fragments bearing identical warheads.^13^ Therefore, to rank covalent inhibitors by affinity, each molecule's inherent reactivity must be considered. Unfortunately, it is difficult to predict electrophile–thiol reactivity trends, such that either computationally expensive QM/DFT calculations must be implemented or low‐throughput experimental techniques performed (for example, NMR or LCMS).^14^ Glutathione (GSH) is widely used for such experiments, but the extent to which it models proteinaceous cysteine reactivity, which is subject to the local protein environment, is unknown. Furthermore, aerobic oxidation of small molecule‐derived thiols makes determination of kinetics challenging.^15^ Therefore, there is an urgent need for methods to screen covalent fragments that relate protein reactivity to intrinsic fragment electrophilicity.

To measure the kinetics of electrophile‐thiol conjugation we developed quantitative irreversible tethering (qIT), a technique which employs fluorogenic thiol quantification (FTQ) in a high‐throughput thiol consumption assay (Figure 1 a). A wide range of FTQ probes have previously been applied in biochemical assays with great success and here we identified 7‐diethylamino‐3‐(4′‐maleimidylphenyl)‐4‐methylcoumarin (CPM) as an ideal choice because of its impressive fluorogenic amplitude, stability in reaction with both small molecule‐ and protein‐derived thiols and its widespread availability.^16^, ^17^, ^18^, ^19^, ^20^ However, using glutathione as a model thiol, aerobic thiol oxidation prevented accurate thiol quantification. Employment of soluble disulfide reducing agents was unsuccessful as they react fluorogenically with FTQ probes (Supporting Information, Figure S1) and operation under anaerobic conditions proved impractical. We solved this problem using an immobilized reducing agent (TCEP‐agarose, 2 % w/v), allowing facile separation by centrifugation before conducting the FTQ step (Figure 1 b).

**Figure 1:**
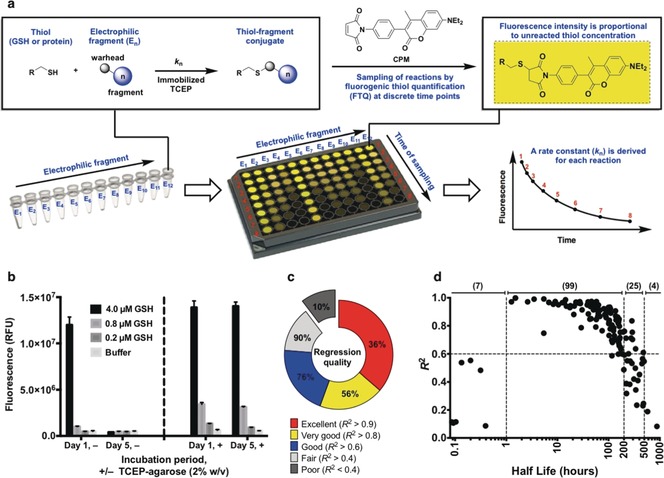
Quantitative irreversible tethering (qIT). a) Assay overview. The target thiol (5 μm) is reacted with electrophilic fragments (0.5 mm) under pseudo‐first‐order conditions in the presence of TCEP‐agarose (2 % w/v). Reaction progress is followed by discrete measurements of residual target thiol concentration using the fluorogenic probe CPM and rate constants are derived from exponential regression analysis. b) TCEP‐agarose prevents aerobic thiol oxidation. Glutathione solutions were stored ±TCEP‐agarose (2 % w/v). Agarose‐beads were separated by centrifugation prior to fluorogenic thiol quantification with CPM after one hour or five days. c) Performance of qIT in determining rate constants for library members in reaction with glutathione is reflected by the coefficient of determination (*R* ^2^) for each exponential regression. d) Accuracy of kinetic modeling as a function of reaction half‐life (*t*_1/2_). Optimum assay performance is achieved when 1>*t*_1/2_>200 hours.

We constructed a 138‐member electrophilic fragment library, predominantly comprising acrylamide warheads but also containing other electrophiles such as chloroacetamides, epoxides and S_N_Ar substrates, where each compound was designed to be largely rule‐of‐three^21^ compliant and possess a unique Bemis–Murcko^22^ framework (Supporting Information, Figure S2). Electrophiles were individually reacted with glutathione under pseudo‐first‐order conditions in the presence of TCEP‐agarose. At eight timepoints over 126 hours, an aliquot of each reaction was quenched into excess CPM. Fluorescence measurements were normalized against a DMSO control and exponential regressions used to determine rate constants. Under these conditions the kinetics could be accurately modeled for reactions with half‐lives (*t*_1/2_) between 1 and 500 hours (mean *R* ^2^=0.79) which accounted for 90 % of the library (average half‐life=132 hours), while the optimum half‐life was between 1 and 200 hours (mean *R* ^2^=0.87) (Figures 1 c and d).

Next we tested whether qIT could also screen electrophilic fragments against proteins. We chose to target Cdk2, which is a clinically important protein in oncology, the activity of which is important for driving cell replication and is dependent upon association with a cyclin protein.^23^ Although Cdk2 possesses three cysteine residues, numerous Cdk2 crystal structures show that only one (C177) is surface‐exposed. Indeed, intact protein mass spectrometry showed Cdk2 to be mono‐modified by CPM while Cdk2(C177A) remained unlabeled (Supporting Information, Figure S3). Significantly, qIT was successful in determining the kinetics of the electrophilic fragment library in reaction with C177 on Cdk2, demonstrating its capability in quantifying reaction kinetics for both small molecule‐ and protein derived‐thiols in high‐throughput (Supporting Information, Table S1).

We then characterized the assay's statistical robustness by determining *Z*′ factors, where for application to high‐throughput screening *Z*′>0.5, 0.6 or 0.7 is generally considered sufficient, good, or excellent, respectively.^24^ Assays against glutathione and Cdk2(WT) were compared to glutamate and Cdk2(C177A) as negative controls. Since qIT relies on consistent time‐dependent measurements, *Z*′ factors were determined at several time points after assay initiation, revealing good‐to‐excellent (0.63<*Z*′<0.74) performance in all cases (Figure 2; Supporting Information, Figure S4).

**Figure 2:**
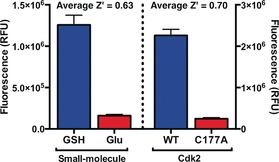
Z′ factor analysis for qIT. Positives=Cdk2(WT) and GSH; negatives=Cdk2(C177A) and glutamate (*n*=72).

The use of glutathione as a control thiol for investigating proteinaceous cysteine reactivity has recently been brought into question.^5^ To explore this, we screened our compound library against a set of seven Cdk2(C177A) mutants, each containing a single surface‐exposed cysteine residue in different settings, including a cryptic hydrophobic binding pocket^25^ (H71C), a protein‐protein interaction interface (S276C) as well as basic (T182C) and acidic (N272C) local environments. Accordingly, we generated a matrix of 1080 rate constants and thence determined the average reactivity of each fragment and each target thiol (Supporting Information, Table S1). Next we performed correlation analysis, comparing fragment reactivity with glutathione (*k*_GSH_) against average fragment reactivity across the seven proteins (*k̄*_protein_). Interestingly, we found strong correlation between the two data sets (Figure 3 a), implying that glutathione can function as an effective control for intrinsic reactivity and suggesting that rate enhancement factor (REF=*k*_protein_/*k*_GSH_) could be used to rank hit ligands. To achieve a false‐discovery rate of less than 2.5 %, we defined rate accelerated and retarded fragments being those with a REF >3 and <0.3, respectively (Supporting Information, Figure S5).

**Figure 3:**
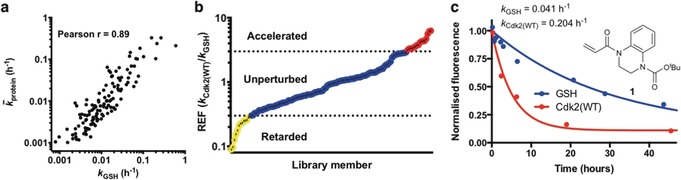
a) Correlation analysis comparing fragment reactivity with glutathione (*k*_GSH_) to average fragment reactivity across seven Cdk2 mutants (*k̄*_protein_). b) Distribution of rate enhancement factors for the covalent fragment library screened against Cdk2(WT). c) DMSO‐Normalized fluorescence data from qIT assay for acrylamide **1** (0.5 mm) in reaction with Cdk2(WT) or glutathione (5 μm) (*n*=2).

Cys177 on Cdk2(WT) is positioned adjacent to the cyclin‐binding interface, and we next investigated whether covalent modification of this site could affect the holoenzyme's activity. Among the fragments that showed accelerated reactivity with Cdk2(WT), acrylamide **1** (REF=5.0) was further investigated because of its impressive proteinaceous selectivity profile (Figures 3 b,c; Supporting Information, Figure S6). Intact protein mass spectrometry confirmed that acrylamide **1** mono‐modifies Cdk2(WT), even in the presence of millimolar glutathione, while Cdk2(C177A) was unaffected (Supporting Information, Figure S7). Interestingly, Cdk2(WT) completely modified with acrylamide **1** exhibited about 83 % inhibition of kinase activity (Figure 4). A series of analogues, synthesized to probe the mechanism of inhibition, revealed that the aromatic ring is crucial to binding specificity while functionalization of the aromatic ring was tolerated for effective labeling and inhibition (Supporting Information, Figures S8 and S9). Interestingly, although substitution of the *tert*‐butyl carbamate for a methyl carbamate (acrylamide **2**) resulted in a similar labeling profile (REF=2.6, *k*_GSH_=0.089 h^−1^, *k*_Cdk2(WT)_=0.233 h^−1^), no significant inhibition was observed.

**Figure 4:**
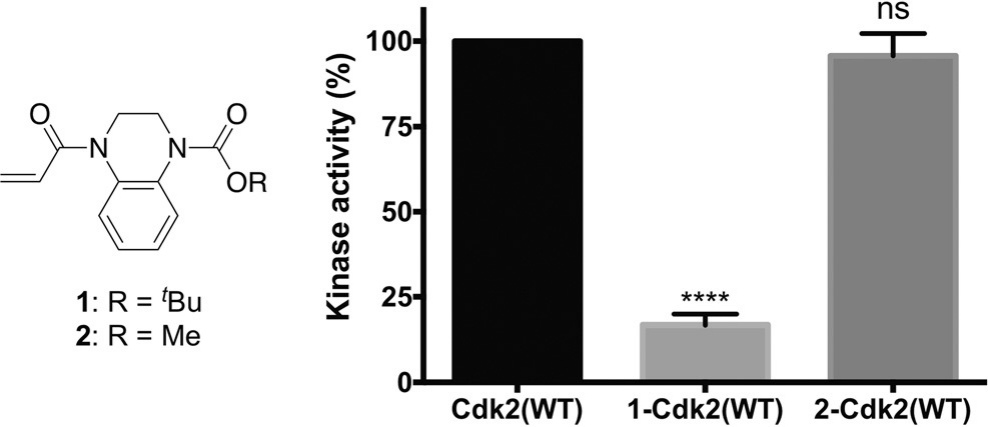
Kinase activity of **1**‐Cdk2(WT) (16.8±3.1 %) and **2**‐Cdk2(WT) (96.6±5.6 %) are reported relative to Cdk2(WT) (*n*=3; error bars and ± denote SEM).

Crystallization of **1**‐Cdk2(WT) showed that the acrylamide had labeled Cys177 with the ligand bound into a shallow pocket adjacent to the cyclin‐binding interface, interacting with Trp227 and Met233 (Figure 5 a). Based on comparison with the structure of Cdk2‐cyclin A2 (PDB: 1FIN), acrylamide **1** points away from the protein–protein interface and so direct hindrance of cyclin binding is unlikely to be the mechanism of Cdk2 inhibition. Although **1**‐Cdk2(WT) showed only minor structural deviations from Cdk2(WT) in the region surrounding the ligand, significant distortion was observed around the αC‐helix (Supporting Information, Figure S11). The αC‐helix is essential for proper formation of the active Cdk2‐cyclin complex and its displacement is implicit in allosteric modulation of various kinases.^26^, ^27^

**Figure 5:**
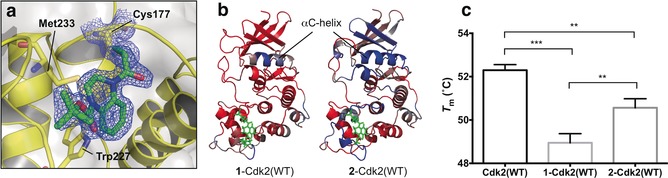
a) Crystal structure of **1**‐Cdk2(WT) (resolution: 1.83 Å, PDB ID: 5OSJ). 2 *F*_o_−*F*_c_ Electron density map (blue) is contoured at 1σ around C177 (yellow) and the ligand (green). b) Molecular dynamics simulations (50 ns) of **1**‐Cdk2(WT) and **2**‐Cdk2(WT) (resolution: 1.72 Å, PDB ID: 5OO0). Atomic flexibility (root mean square fluctuations) was compared to a similar simulation for Cdk2(WT) (ligands in green, flexibility: red=increase; blue=decrease and grey=unperturbed). c) *T*_m_ were determined by TdCD (*n*=3; error bars=SD). **, *** and **** denote *P*<0.01, 0.001 and 0.0001 respectively in two‐tailed T‐test).

To model how acrylamide **1** was affecting enzymatically relevant changes to the structure and dynamics of Cdk2, molecular dynamics (MD) simulations were performed. In a 50 ns MD run, **1**‐Cdk2(WT) showed increased flexibility compared to Cdk2(WT) across the entire structure, with the exception of the αC‐helix, suggesting a general destabilization of the protein which is supported by an experimentally observed drop in melting temperature (Δ*T*_m_=−3.4 °C) (Figures 5 b and c).

Interestingly, while crystallization of **2**‐Cdk2(WT) revealed that acrylamides **1** and **2** adopt similar binding conformations, the representative structure from the MD trajectory of **1**‐Cdk2(WT) is closer to Cdk2(WT) at the αC‐helix, with only a modest change in global flexibility (Supporting Information, Figure S12). Indeed, **2**‐Cdk2(WT) is only moderately destabilized (Δ*T*_m_=−1.7 °C) relative to Cdk2(WT), which correlates with its weak inhibition.

Although several Cdk2‐targeting drugs have failed in clinical trials as chemotherapeutics, these ATP‐competitive inhibitors had only moderate selectivity for Cdk2.^28^ Indeed, owing to the similarity of their ATP‐binding sites, Cdk1 has proved especially hard to eliminate as an off‐target and the resulting Cdk1‐dependent toxicity narrows the therapeutic window (Figure 6 b). Conducting a kinome‐wide sequence alignment revealed that the pocket surrounding Cys177 (Cdk2 residues 169–298) is present within much of the CMGC kinase group, with Cdks showing the closest similarity. However, it is noteworthy that only Cdk2 has a cysteine at the position equivalent to 177 and we therefore anticipated that the strategy of Cys177 modification would enable unprecedented specificity for Cdk2 inhibition (Figure 6 a).^29^ Indeed, acrylamide **1** failed to inhibit Cdk1‐cyclin A2 in vitro after prolonged exposure (24 hours; Figure 6 c).

**Figure 6:**
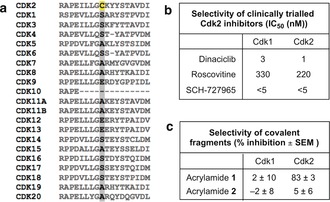
a) Sequence alignment of Cdk family. Cys177 is highlighted in yellow. b) Published selectivity profiles of selected Cdk2 inhibitors.^29^ c) Active Cdk1/Cdk2:cyclin A2 was incubated with acrylamides **1** and **2** (0.5 mm) for 24 hours and then their in vitro kinase activity was measured (*n*=3).

To evaluate whether this in vitro activity would translate into in‐cell target engagement, acrylamide **3** (REF_Cdk2(WT)_=5.6, *k*_GSH_=0.033 h^−1^, *k*_Cdk2(WT)_=0.185 h^−1^) was synthesized as a target engagement probe, containing an alkyne handle for bio‐orthogonal ligation. HeLa cells were treated with probe **3** and protein lysates subjected to copper(I) catalyzed azide–alkyne cycloaddition (CuAAC)^30^ to affect biotin conjugation. After pull‐down with Neutravidin beads, western blot analysis revealed a concentration‐dependent enrichment of Cdk2, while Cdk1 was not isolated (Figure 7). These data demonstrate that, by virtue of targeting a unique cysteine, these cell‐permeable acrylamides provide a starting point for a novel class of Cdk2‐selective probes.

**Figure 7:**
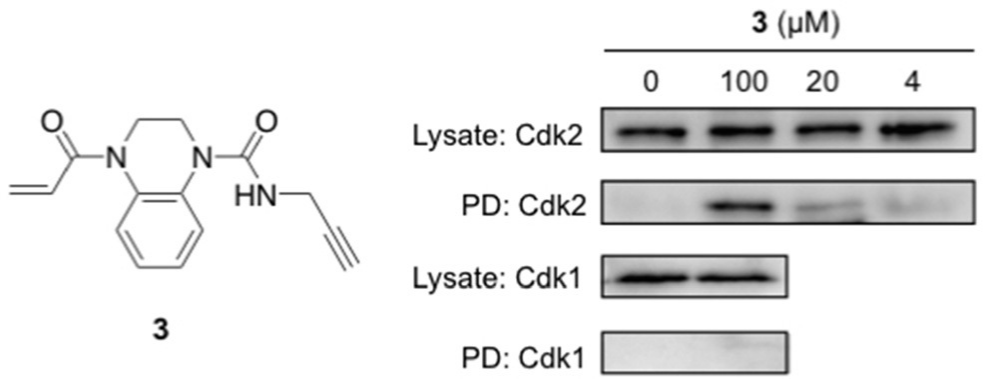
HeLa cells were treated with **3** for 6 hours. After tagging with biotin‐azide, Neutavidin pulldown was performed. Western blot analysis (anti‐Cdk2 and Cdk1) was performed before (Lysate) and after pulldown (PD).

In conclusion, we have established a platform for screening electrophilic fragments with heterogeneous reactivity by virtue of parallel kinetic analysis of target and control thiols. Using this approach, we were able to rapidly identify and validate hit‐fragments against a shallow pocket. Moreover, we have shown how covalent modification of a unique cysteine residue can enable allosteric modulation of Cdk2 activity with unprecedented selectivity. The assay is directed to a predefined site on the target protein and is independent of the protein function, making it ideal to screen for inhibitors of protein–protein interactions, for example. We envisage that this simple and cost effective approach to target‐directed covalent ligand discovery will accelerate the development of new chemical probes and expand the size of the druggable proteome.

## Conflict of interest

G.B.C., S.M., A.A., and D.J.M. are co‐inventors on a patent application covering qIT: PCT/GB2017/052456.

## Supporting information

As a service to our authors and readers, this journal provides supporting information supplied by the authors. Such materials are peer reviewed and may be re‐organized for online delivery, but are not copy‐edited or typeset. Technical support issues arising from supporting information (other than missing files) should be addressed to the authors.

Supplementary
