# Supplementary material for: High‐Throughput Kinetic Analysis for Target‐Directed Covalent Ligand Discovery
Source: Angew Chem Int Ed Engl. 2018 Mar 26;57(19):5257–61. doi: 10.1002/anie.201711825 (PMC5947712; doi:10.1002/anie.201711825)
Supplement: Supplementary file 1 — Supplementary [file ANIE-57-5257-s001.pdf]

Angewandte Chemie
Eine Zeitschrift der Gesellschaft Deutscher Chemiker

Supporting Information

High-Throughput Kinetic Analysis for Target-Directed Covalent Ligand Discovery

*Gregory B. Craven, Dominic P. Affron, Charlotte E. Allen, Stefan Matthies, Joe G. Greener, Rhodri M. L. Morgan, Edward W. Tate, Alan Armstrong, and David J. Mann**

anie_201711825_sm_miscellaneous_information.pdf

Table of Contents

Supplementary Results ... 2
  Supplementary Figures ... 2
  Supplementary Tables ... 2

Supplementary Methods ... 9
  A. Chemical synthesis ... 9
  B. Synthesis and characterization of compounds ... 11
  C. Library design ... 27
  D. Rate determination by qIT ... 27
  E. Z' Factor determination ... 27
  F. Protein expression ... 27
  G. Primers ... 27
  H. Protein purification ... 28
  I. Acrylamide labelling of Cdk2 for crystallography, TdCD, kinase assays and intact-protein mass spectrometry ... 28
  J. Intact-protein mass spectrometry ... 28
  K. Crystallography ... 28
  L. Molecular dynamics simulation ... 29
  M. Data collection and structure determination ... 29
  N. Cdk2 phosphorylation ... 29
  O. Kinase assay ... 29
  P. TdCD ... 29
  Q. *In cell* target engagement assay ... 29
  R. Immunoblot analysis ... 30

Supplementary Reference ... 31

Supplementary Results

Supplementary Figures

Figure S1: Phosphine-based reducing agents react stoichiometrically with CPM
Figure S2: Chemical structures and physiochemical properties of fragment library
Figure S3: Cdk2(WT) is mono-modified by excess CPM
Figure S4: Full Z' factor analysis
Figure S5: Full correlation analysis
Figure S6: Acrylamide 1 selectivity profile
Figure S7: Cdk2(WT) is mono-modified by acrylamide **1**
Figure S8: qIT Data for acrylamide **2**
Figure S9: Covalent-SAR analysis of Cdk2(WT) inhibitors
Figure S10: Dose response curves for acrylamides **1** and **2**
Figure S11: Acrylamide 1 effects a distortion of the αC-helix
Figure S12: Crystal structure of **2**-Cdk2(WT) and molecular dynamics simulations
Figure S13: Target-engagement experiments

Supplementary Tables

Table S1: Matrices of rate constants and REFs (see separate excel file)
Table S2: Data collection and refinement statistics for 5OSJ
Table S3: Data collection and refinement statistics for 5OO0

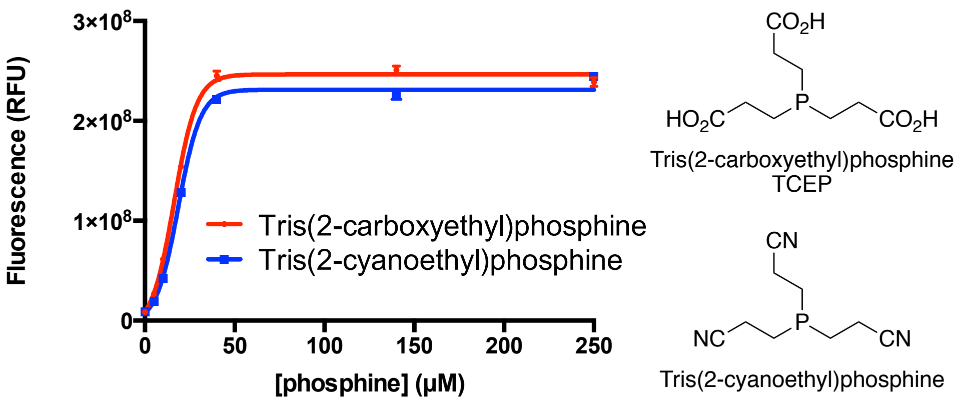

Supplementary Figure S1 | Phosphine-based reducing agents react stoichiometrically with CPM. Phosphine reducing agents were titrated against CPM (50 μM) in 100 mM phosphate buffer (pH 7.5) and incubated at room temperature for 60 minutes. Fluorescence (excitation/emission: 384/470 nm) was measured in triplicate.

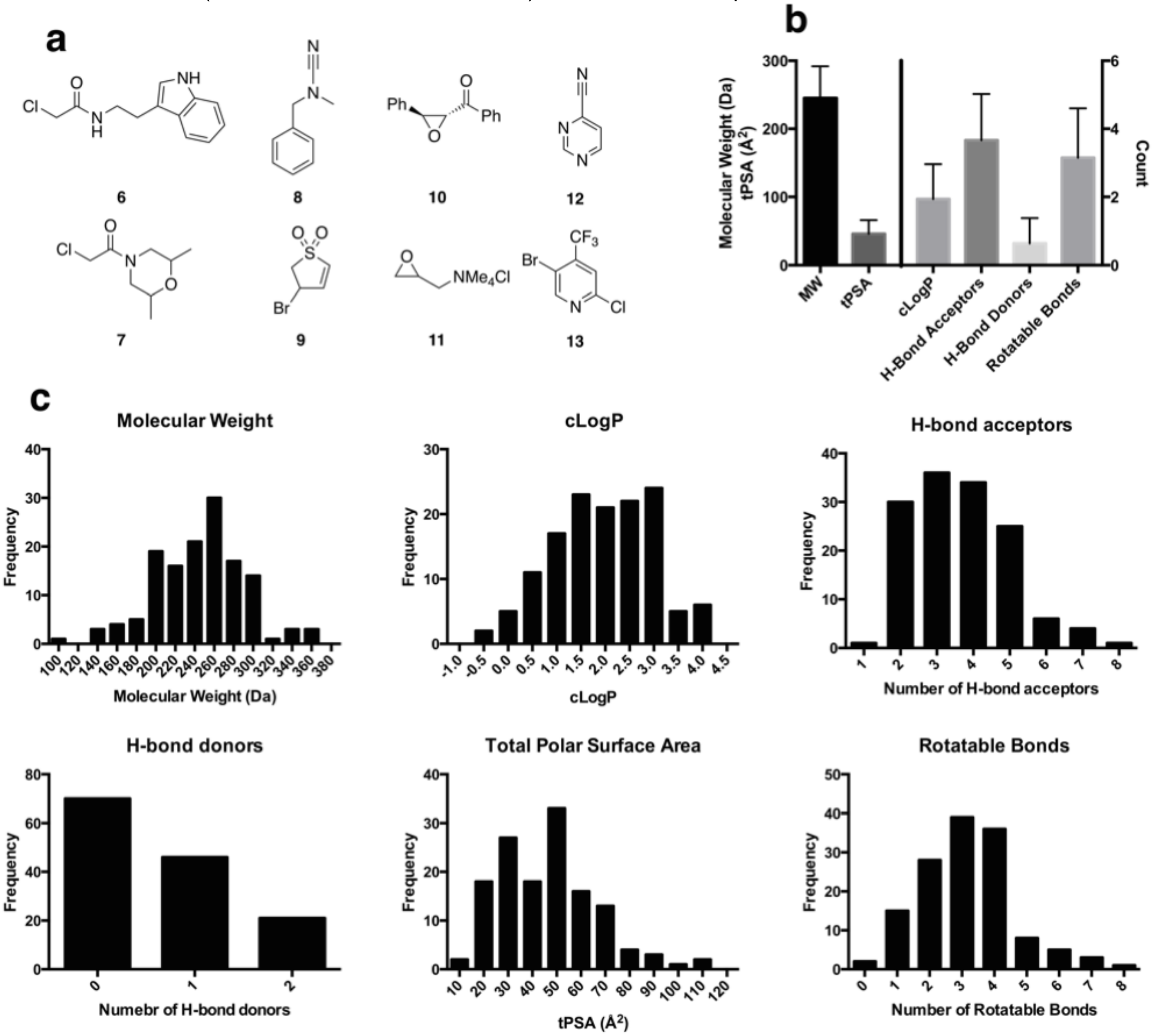

Supplementary Figure S2 | Chemical structures and physiochemical properties of fragment library. (**a**) Selected compounds from the library illustrating variety of warheads: chloroacetamides (**6** and **7**), cyanamide (**8**), vinylsulfone (**9**), epoxide (**10** and **11**), arylnitrile (**12**) and chloropyridine (**13**). (**b**) Average physicochemical properties of library (error bars = SD). (**c**) Distribution of individual physicochemical properties of library.

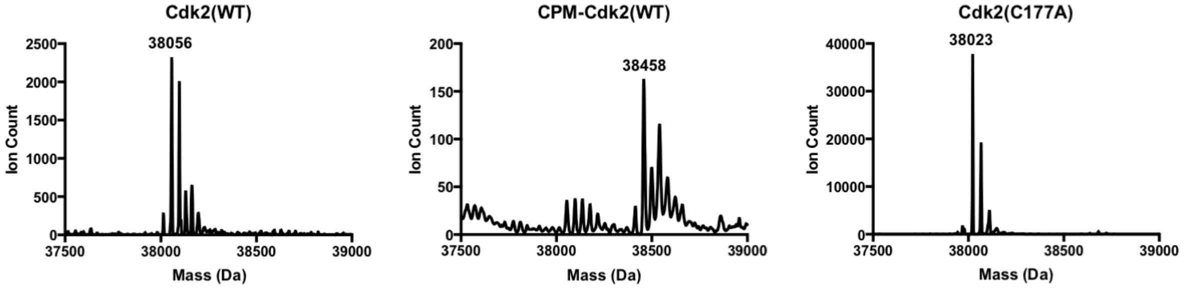

Supplementary Figure S3 | Cdk2(WT) is mono-modified by excess CPM. Cdk2(WT)/Cdk2(C177A) (10 μM) was incubated with CPM (50 μM) in 100 mM phosphate buffer (pH 7.5) and incubated at room temperature for one hour. Intact protein mass spectra were recorded before and after incubation and show the expected mass increase of 402 Da for Cdk2(WT) but no change for Cdk2(C177A).

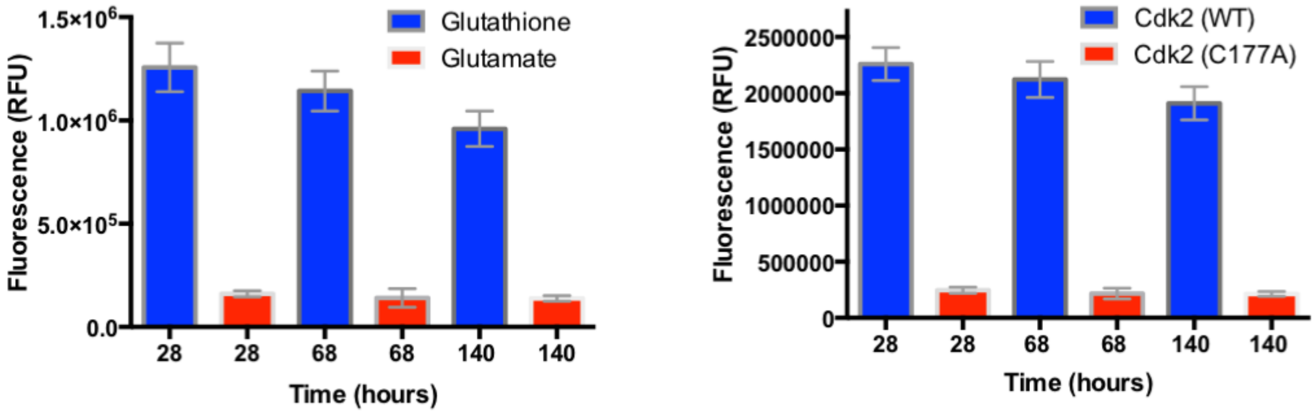

Time	Average	28 h	68 h	140 h
Z' GSH	0.63	0.63	0.64	0.64

Time	Average	28 h	68 h	140 h
Z' Cdk2(WT)	0.70	0.74	0.67	0.70

Supplementary Figure S4 | Full Z' factor analysis. Thiol solutions (positives = Cdk2(WT) and glutathione; negatives = Cdk2(C177A) and glutamate) were incubated with TCEP-agarose (2% v/v). After 28, 68 and 140 hours, fluorogenic quantifications were performed for each thiol (n = 72). Z' factors were calculated using the formula $Z' = 1 - (3(\sigma_p + \sigma_n)/|\mu_p - \mu_n|)$, where σ_p = standard deviation of positives, σ_n = standard deviation of negatives, μ_p = mean of positives, μ_n = mean of negatives.

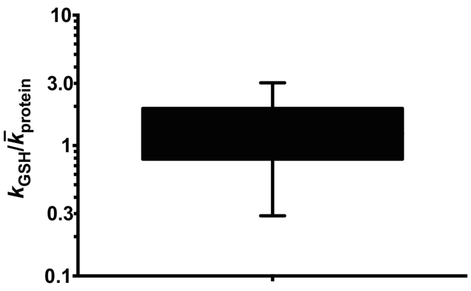

Supplementary Figure S5 | Full correlation analysis. Box and whisker plot (the box extends from the 25th to 75th percentiles and the whiskers from the 2.5th to 97.5th percentile) of $k_{GSH}/\bar{k}_{protein}$. REF cutoffs, 3.0 and 0.3 for rate acceleration and retardation respectively, were based on the 2.5th to 97.5th percentiles.

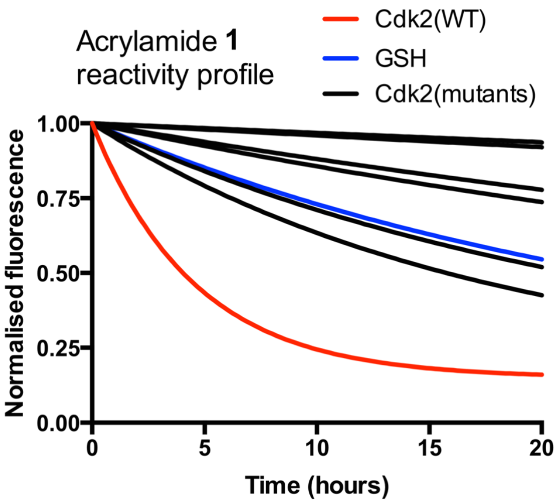

Supplementary Figure S6 | Acrylamide 1 selectivity profile. Exponential regressions from kinetic analysis of acrylamide **1** (0.5 mM) in reaction with various thiols (5 μM).

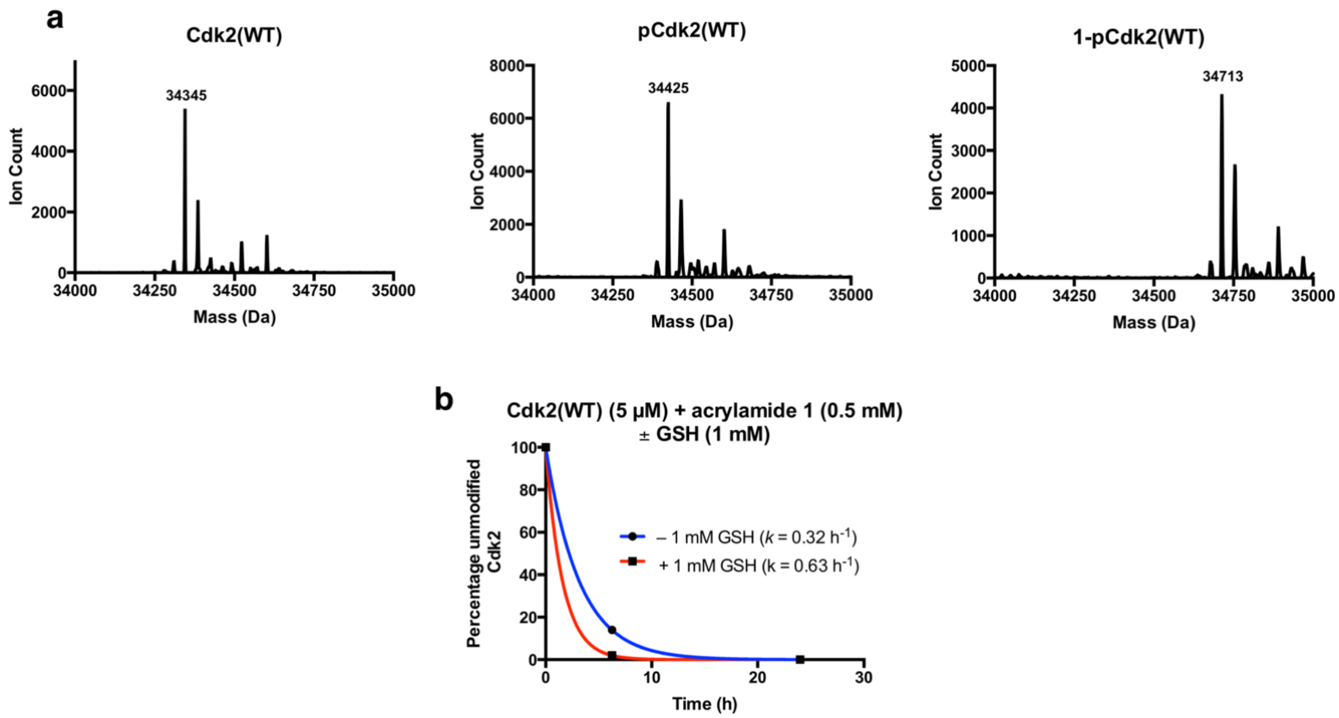

Supplementary Figure S7 | Cdk2(WT) is mono-modified by acrylamide 1. (**a**) Cdk2(WT) was phosphorylated with CAK1. pCdk2(WT) (5 μM) was then incubated with acrylamide **1** (0.5 mM) in 100 mM phosphate buffer (pH 8.0) at 4 ˚C for 18 hours. Intact-protein mass spectra show complete phosphorylation (ΔMW = 80 Da) and complete acrylamide labeling (ΔMW = 288 Da). (**b**) Cdk2(WT) (5 μM) was incubated with acrylamide **1** (0.5 mM) in 100 mM phosphate buffer (pH 8.0) in the presence or absence of GSH (1 mM) at room temperature. The degree of labelling was monitored by intact-protein mass spectrometry after 6.25 and 24 h. The presence of 1 mM GSH was found to slow the rate of Cdk2 modification by a factor of ~2.

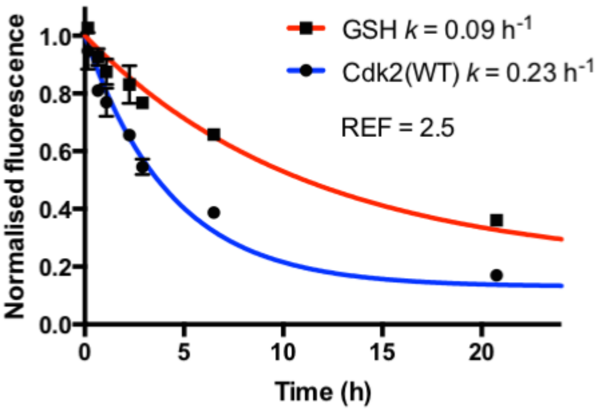

Supplementary Figure S8 | qIT Data for acrylamide 2. DMSO-Normalised fluorescence data from qIT assay for acrylamide **2** (0.5 mM) in reaction with Cdk2(WT) or glutathione (5 μM).

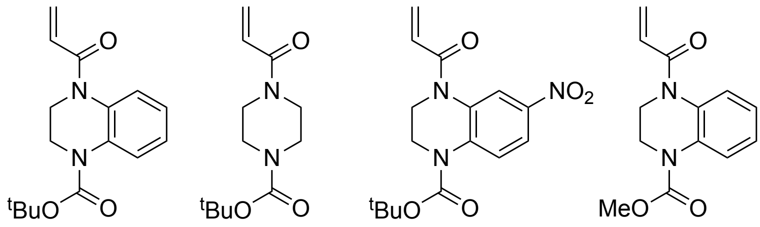

1 **4** **5** **2**

Compound	$k_{Cdk2(WT)}$ (h^{-1})	k_{GSH} (h^{-1})	$REF_{Cdk2(WT)}$	Kinase activity (%) ± s.e.m (n = 3)	T_m (˚C) ± s.e.m (n = 3)	ΔT_m (˚C) (P value)
**1**	0.204	0.041	5.0	16.8 ± 3.1	48.95 ± 0.25	–3.35 (0.0003)
**4**	0.003	0.017	0.42	N/D	N/D	N/D
**5**	1.603	0.596	2.7	20.0 ± 0.8	50.08 ± 0.55	–2.22 (0.0178)
**2**	0.233	0.089	2.6	96.6 ± 5.6	50.56 ± 0.24	–1.74 (0.0036)

Supplementary Figure S9 | Covalent-SAR analysis of Cdk2(WT) inhibitors. Compounds (0.5 mM) were reacted with glutathione and Cdk2(WT) (5 μM). The kinetics were monitored by qIT and rate enhancement factors calculated. *In vitro* kinase activity is reported relative to Cdk2(WT). The presence of the aromatic ring is crucial to binding specificity, with *N*-Boc piperazine **4** giving a REF of only 0.42, while functionalization of the aromatic ring is reasonably tolerated for effective labeling and inhibition (acrylamide **5**: REF = 2.69, kinase inhibition = 80%). Substitution of the *tert*-butyl carbamate for a methyl carbamate, to give acrylamide **2,** results in a similar labeling profile (REF = 2.63), however no significant inhibition is observed. T_m were determined by TdCD. N/D = not determined.

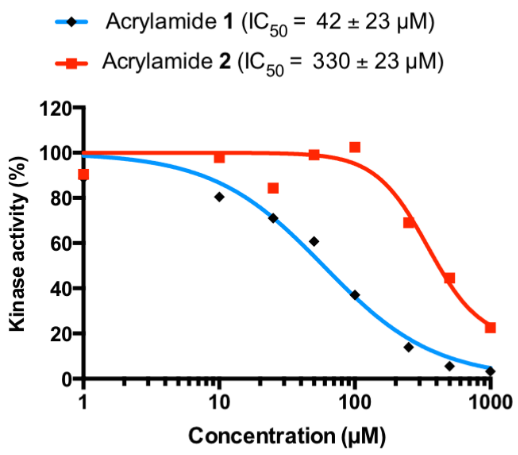

Supplementary Figure S10 | Dose response curves for acrylamides 1 and 2. pCdk2(WT) (0.5 µM) was incubated with acrylamides **1** or **2** (1 – 1000 µM) in 100 mM phosphate buffer (pH 8.0) at 37 ˚C for 18 hours. The kinase activity was measured relative to the DMSO control and IC_{50} values calculated using sigmoidal regression analysis (data is representative of 2 repeats, ± denote SD).

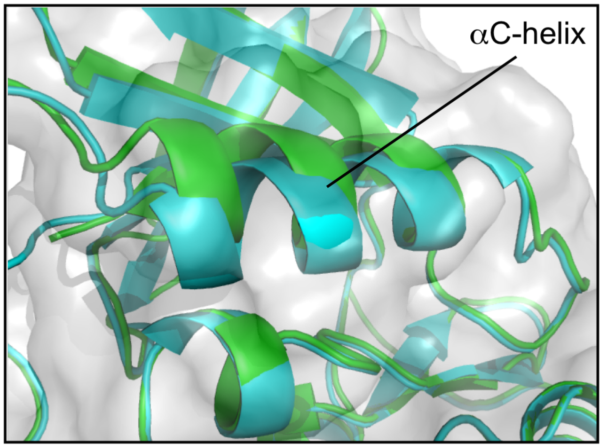

Supplementary Figure S11 | Acrylamide 1 effects a distortion of the αC-helix. Overlay of **1**-Cdk2(WT) (cyan) and Cdk2(WT) (green, PDB: 4EK3) crystal structures around the αC-helix.

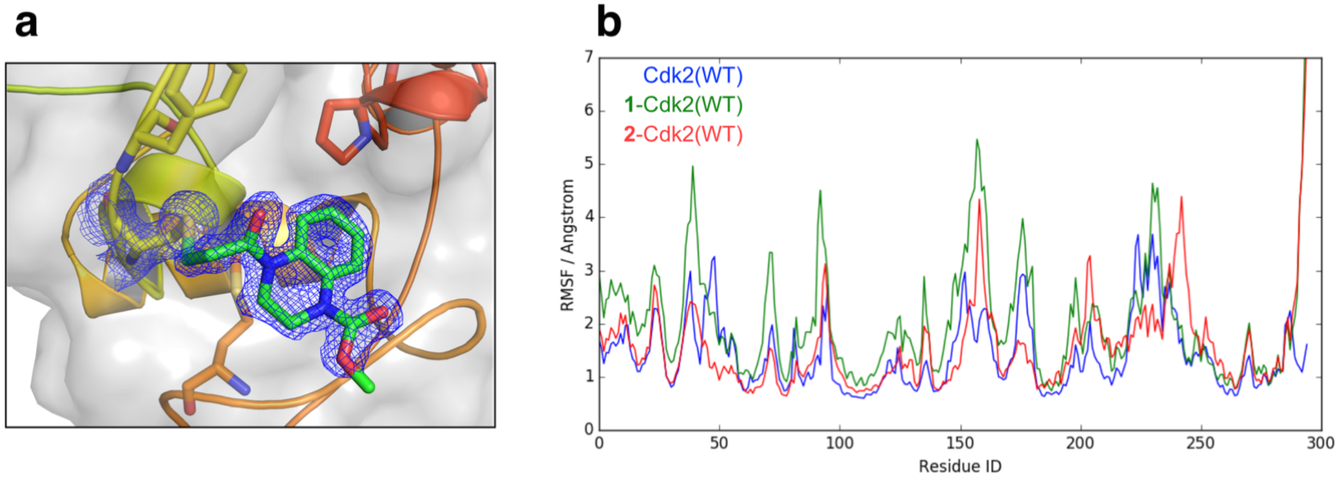

Supplementary Figure S12| Crystal structure of 2-Cdk2(WT) and molecular dynamics simulations. (**a**) Crystal structure of **2**-Cdk2(WT) (resolution: 1.60 Å, PDB ID: 5OO0). The $2F_o–F_c$ electron density map (blue) is contoured at 1σ around Cys177 (yellow) and the ligand (green). (b) Graph of root mean squared fluctuation by residue for Cdk2(WT) (blue), **1**-Cdk2(WT) (green) and **2**-Cdk2(WT) (red).

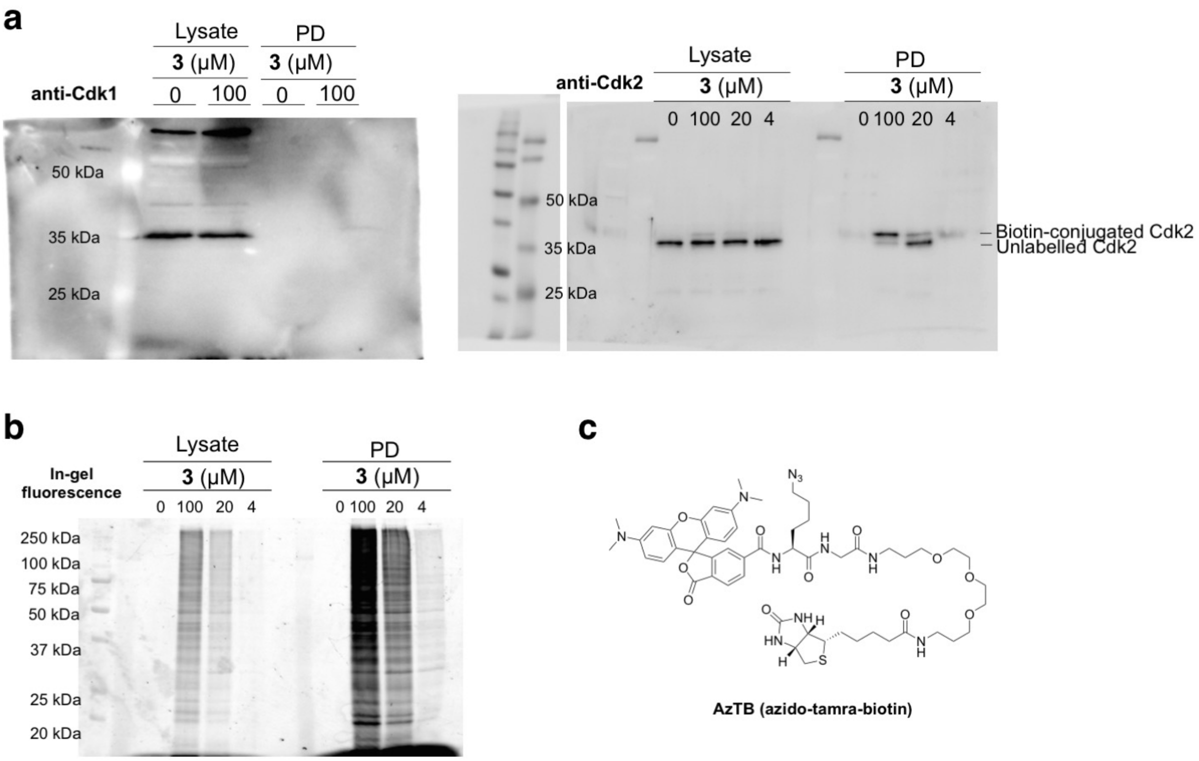

Supplementary Figure S13 | Target-engagement experiments. HeLa cells were treated with **3** for 6 hours. After tagging with AzTB, Neutavidin pulldown was performed. (**a**) Western blot analysis (anti-Cdk2 and Cdk1) was performed before (Lysate) and after pulldown (PD). The CuAAC biotin conjugation causes a resolvable electrophoretic mobility shift. Some unlabbled Cdk2 appears after pull-down due to background binding to the Neutravidin beads. (**b**) In-gel fluorescence (ex/em = 546/579 nm) was performed on samples from before (Lysate) and after pulldown (PD). (**c**) Chemical structure of AzTB.

Table S2. Data collection and refinement statistics for 5OSJ

PDB: 5OSJ	1-Cdk2(WT)
**Wavelength**	0.9763
**Resolution range**	50.30 - 1.83 (1.877 - 1.83)
**Space group**	P 21 21 21
**Unit cell**	53.916 71.111 72.25 90 90 90
**Total reflections**	50215 (4848)
**Unique reflections**	23782 (1733)
**Multiplicity**	2.0 (2.0)
**Completeness (%)**	100 (99)
**Mean I/sigma(I)**	17.20 (3.70)
**Wilson B-factor**	19.39
**R-merge**	0.02344 (0.2051)
**R-meas**	0.03315 (0.2901)
**CC1/2**	0.999 (0.858)
**CC***	1 (0.961)
**Reflections used in refinement**	25116 (2430)
**Reflections used for R-free**	1335 (112)
**R-work**	0.1738 (0.267)
**R-free**	0.2220 (0.297)
**CC(work)**	0.955 (0.856)
**CC(free)**	0.925 (0.737)
**Number of non-hydrogen atoms**	2618
**macromolecules**	2310
**ligands**	21
**Protein residues**	292
**RMS(bonds)**	0.021
**RMS(angles)**	1.95
**Ramachandran favored (%)**	99

**Ramachandran allowed (%)**	0.69
**Ramachandran outliers (%)**	0
**Rotamer outliers (%)**	1.6
**Clashscore**	3.23
**Average B-factor**	22.70
**macromolecules**	21.52
**ligands**	23.92
**solvent**	32.14

Statistics for the highest-resolution shell are shown in parentheses.

Table S3. Data collection and refinement statistics for 5OO0

**PDB: 5OO0**	**2-Cdk2(WT)**
**Wavelength**	
**Resolution range**	42.9 - 1.6 (1.642 - 1.6)
**Space group**	P 21 21 21
**Unit cell**	53.513 71.024 72.152 90 90 90
**Total reflections**	72845 (7071)
**Unique reflections**	34806 (2502)
**Multiplicity**	2.0 (2.0)
**Completeness (%)**	99 (98)
**Mean I/sigma(I)**	21.87 (7.99)
**Wilson B-factor**	17.41
**R-merge**	0.015 (0.6418)
**R-meas**	0.02121 (0.9076)
**CC1/2**	0.999 (0.986)
**CC***	1 (0.996)
**Reflections used in refinement**	36563 (3553)
**Reflections used for R-free**	1761 (140)
**R-work**	0.17229 (0.190)
**R-free**	0.20031 (0.239)
**CC(work)**	0.965 (0.798)
**CC(free)**	0.942 (0.753)
**Number of non-hydrogen atoms**	2625
**macromolecules**	2289
**ligands**	18
**Protein residues**	288
**RMS(bonds)**	0.027
**RMS(angles)**	2.42
**Ramachandran favored (%)**	99
**Ramachandran allowed (%)**	1.4
**Ramachandran outliers (%)**	0
**Rotamer outliers (%)**	1.2
**Clashscore**	3.26
**Average B-factor**	22.59
**macromolecules**	21.25
**ligands**	24.71
**solvent**	32.08

Statistics for the highest-resolution shell are shown in parentheses.

Supplementary Methods

A. Chemical synthesis

All non-aqueous reactions were carried out under an inert atmosphere (argon) with flame-dried glassware, using standard techniques. Anhydrous solvents were obtained by filtration through drying columns (DMF, CH_2Cl_2, THF).

Flash column chromatography was performed using 230-400 mesh silica, with the indicated solvent system according to standard techniques. Analytical thin-layer chromatography (TLC) was performed on precoated aluminium-backed silica gel plates. Visualisation of the developed chromatogram was performed by UV absorbance (254 nm) and/or stained with aqueous potassium permanganate solution, aqueous ceric ammonium molybdate, or a ninhydrin solution in ethanol.

Nuclear magnetic resonance spectra were recorded on 400 MHz or 500 MHz spectrometers. Chemical shifts for ^{1}H NMR spectra are recorded in parts per million from tetramethylsilane with the residual protic solvent resonance as the internal standard (chloroform: δ 7.27 ppm, methanol: δ 3.31 ppm). Data are reported as follows: chemical shift (multiplicity [s = singlet, d = doublet, t = triplet, m = multiplet and br = broad], coupling constant (in Hz), integration). ^{13}C NMR spectra are recorded with complete proton decoupling. Chemical shifts are reported in parts per million from tetramethylsilane with the solvent resonance as the internal standard ($^{13}CDCl_3$: δ 77.0 ppm, $^{13}CD_3OD$: δ 49.0 ppm). Assignments of ^{1}H and ^{13}C spectra were based upon the analysis of δ and *J* values, as well as DEPT, COSY, HMBC, HSQC and nOe experiments where appropriate.

Commercial reagents were used as supplied, or purified by standard techniques where necessary. Compounds **1** and **6**–**13** were used as commercially supplied.

B. Synthesis and characterization of compounds

1-(3,4-Dihydroquinoxalin-1(2*H*)-yl)-2-(phenylselanyl)propan-1-one (S-A)

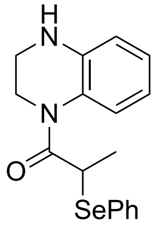

Oxalyl chloride (254 μL, 3.00 mmol) was added dropwise to a solution of 2-(phenylselanyl)propanoic acid (687 mg, 3.00 mmol) in THF (10 mL) at 0 ºC. DMF (2 drops) was added and the reaction mixture was allowed to warm to rt for 2 h. This solution was then added dropwise to a solution of NEt_3 (627 μL, 4.50 mmol) and 1,2,3,4-tetrahydroquinoxaline (403 mg, 3.00 mmol) in THF (10 mL). The reaction mixture was allowed to stir at rt for 18 h. Saturated aqueous $NaHCO_3$ (50 mL) was added, and the resulting mixture was extracted with CH_2Cl_2 (3 × 50 mL). The organic layers were combined, dried over Na_2SO_4, filtered, and the solvent was removed under reduced pressure. The resulting crude material was purified by flash column chromatography (10% grading to 30% EtOAc/pentane), which afforded 1-(3,4-dihydroquinoxalin-1(2*H*)-yl)-2-(phenylselanyl)propan-1-one **S-A** (788 mg, 76%) as a white solid.

m.p = 127–129 ºC ($CHCl_3$)

R_f 0.33 (25% EtOAc/pentane);

ν_{max} (film)/cm^{-1} 3356, 3056, 2939, 2863, 1633, 1603, 1503, 1391, 1323, 738;

^{1}H NMR (400 MHz, $CDCl_3$) δ 7.41–7.32 (m, 2 H), 7.30–7.24 (m, 1 H), 7.23–7.15 (m, 2 H), 7.07–6.97 (m, 2 H), 6.65–6.56 (m, 2 H), 4.71–4.58 (m, 1 H), 4.32–4.19 (m, 1 H), 4.15–4.01 (m, 1 H), 3.55–3.25 (m, 3 H), 1.69 (d, *J* = 6.9 Hz, 3 H);

^{13}C NMR (101 MHz, $CDCl_3$) δ 172.6, 138.3, 134.9 (2 × C_{Ar}), 128.9 (2 × C_{Ar}), 128.4, 127.9, 126.8, 124.4, 124.1, 116.1, 114.6, 42.1, 39.2, 36.7, 19.0;

HRMS (ESI$^+$) *m/z* Calculated for $C_{17}H_{19}N_2O^{80}Se^+$ $[M+H]^+$ 347.0663; Found 347.0679 (Δ +4.6 ppm).

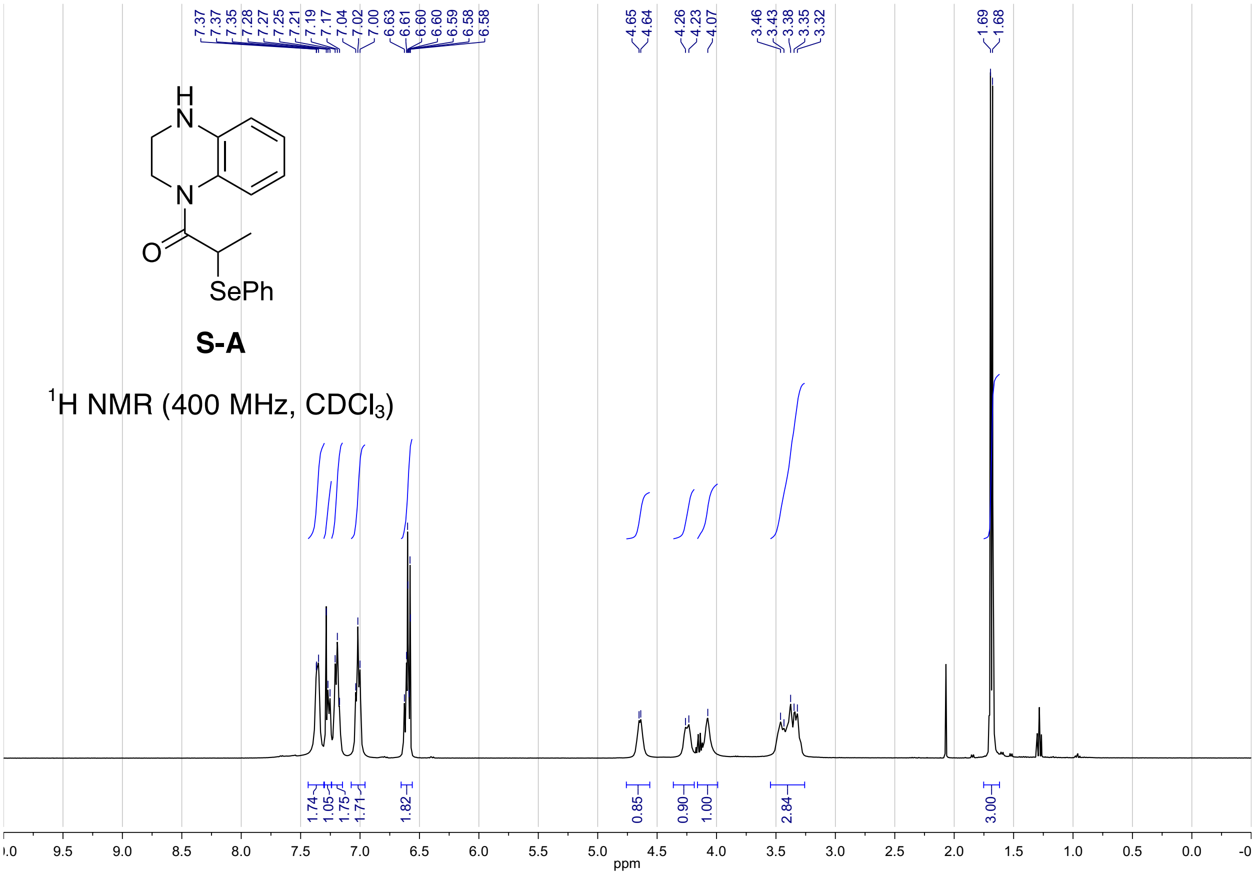

S-A

^{1}H NMR (400 MHz, $CDCl_3$)

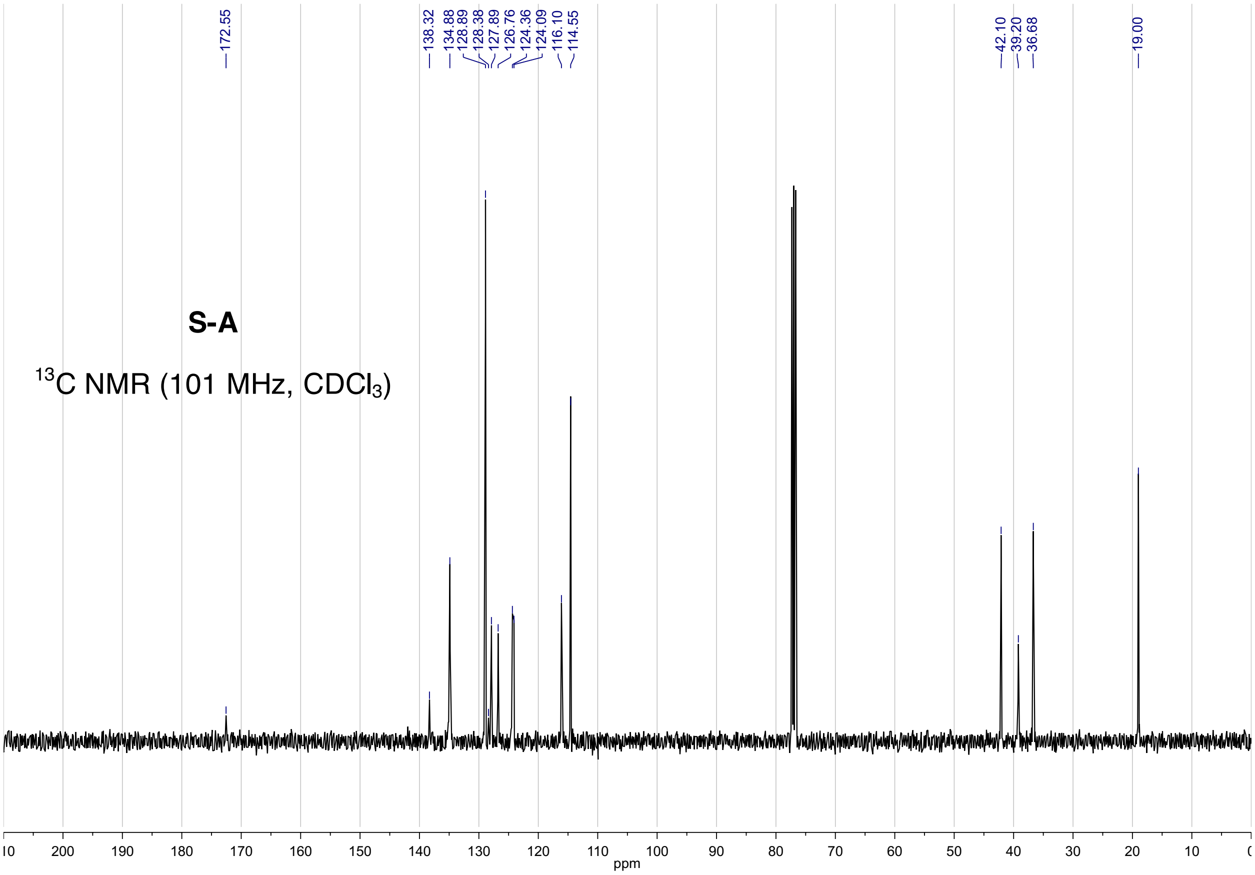

S-A

^{13}C NMR (101 MHz, $CDCl_3$)

Methyl 4-(2-(phenylselanyl)propanoyl)-3,4-dihydroquinoxaline-1(2*H*)-carboxylate (S-2)

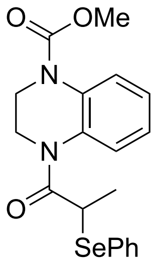

Methyl chloroformate (58 μL, 0.75 mmol) was added dropwise to a solution of potassium carbonate (138 mg, 1.00 mmol) and 1-(3,4-dihydroquinoxalin-1(2*H*)-yl)-2-(phenylselanyl)propan-1-one **S-A** in DMF (1.0 mL) at 0 °C. The resulting solution was then heated to 50 °C for 3 h. The reaction mixture was allowed to cool to rt, then water (15 mL) and CH_2Cl_2 (15 mL) were added and the phases separated. The organic layer was collected, and the aqueous layer was extracted with further CH_2Cl_2 (2 × 15 mL). The combined organic layers were dried over Na_2SO_4 and filtered, then the solvent was removed under reduced pressure. The resulting crude material was then purified by flash column chromatography (15% grading to 20% EtOAc/pentane), which gave methyl 4-(2-(phenylselanyl)propanoyl)-3,4-dihydroquinoxaline-1(2*H*)-carboxylate **S-2** (75 mg, 74%) as a colourless oil.

R_f 0.36 (25% EtOAc/pentane);

ν_{max} (film)/cm^{-1} 2953, 1707, 1651, 1497, 1437, 1390, 1328, 1216, 1191, 1139, 1060, 736, 692;

^{1}H NMR (400 MHz, $CDCl_3$) δ 7.03–7.91 (m, 1 H), 7.51 (d, *J* = 7.5 Hz, 2 H), 7.26–7.03 (m, 5 H), 7.01 (td, *J* = 7.6, 1.4 Hz, 1 H), 4.47–4.36 (m, 1 H), 4.30–4.16 (m, 1 H), 3.84–3.77 (m, 1 H), 3.83 (s, 3 H), 3.75–3.61 (m, 1 H), 3.60–3.45 (m, 1 H), 1.68–1.63 (m, 3 H);

^{13}C NMR (101 MHz, $CDCl_3$) δ 171.8, 154.5, 135.2 (2 × C_{Ar}), 133.0, 131.6, 128.9 (2 × C_{Ar}), 128.2, 127.8, 126.6, 124.0, 123.7, 123.5, 53.1, 46.8, 42.2, 36.4, 18.8;

HRMS (ESI$^+$) *m/z* Calculated for $C_{19}H_{21}N_2O_3{}^{80}Se^+$ [M+H]$^+$ 405.0717; Found 405.0717 (Δ 0.0 ppm).

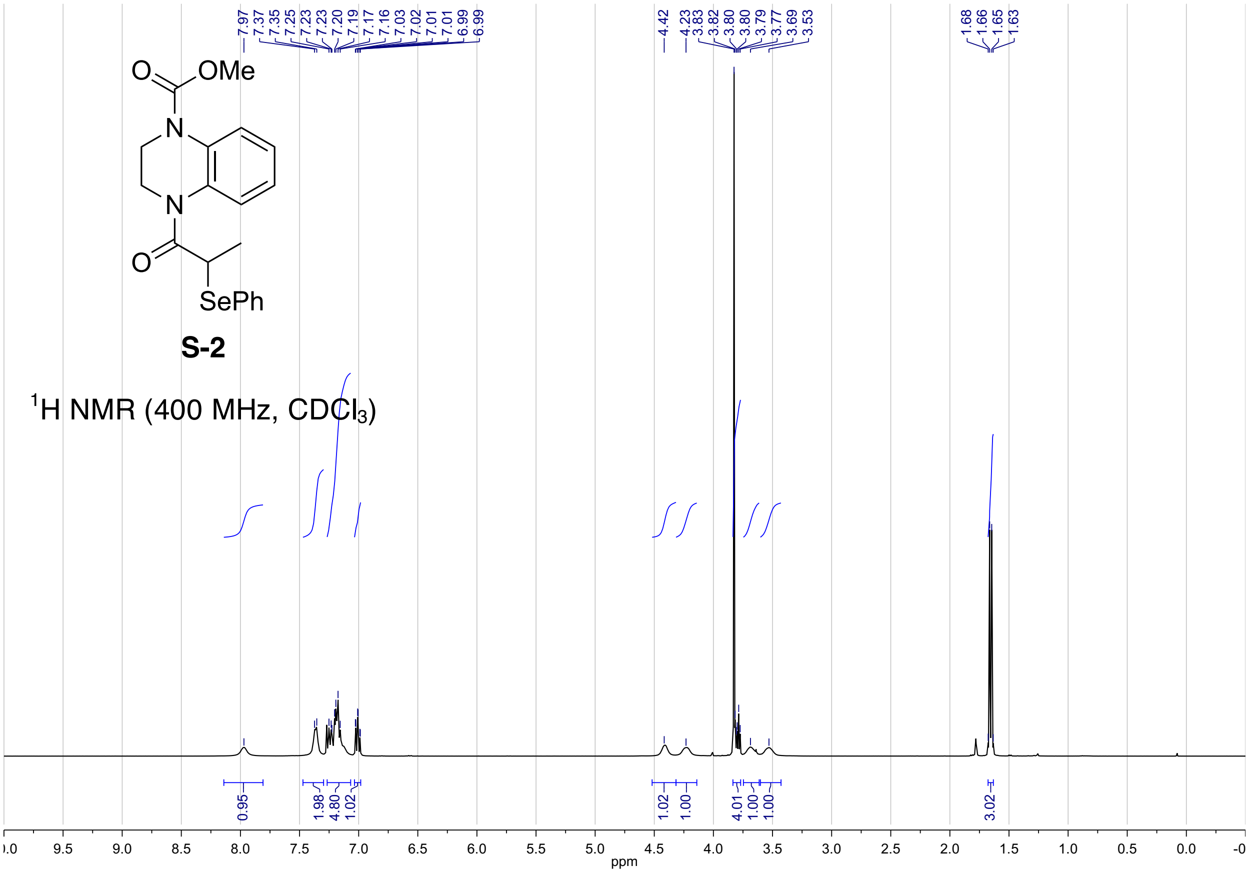
7.97 7.37 7.35 7.25 7.23 7.23 7.20 7.19 7.17 7.16 7.03 7.02 7.01 7.01 6.99 6.99
4.42 4.23 3.83 3.82 3.80 3.80 3.79 3.77 3.69 3.53
1.68 1.66 1.65 1.63
O
OMe
N
N
O
SePh
S-2
^{1}H NMR (400 MHz, $CDCl_3$)
0.95 1.98 4.80 1.02 1.02 1.00 4.01 1.00 1.00 3.02
ppm

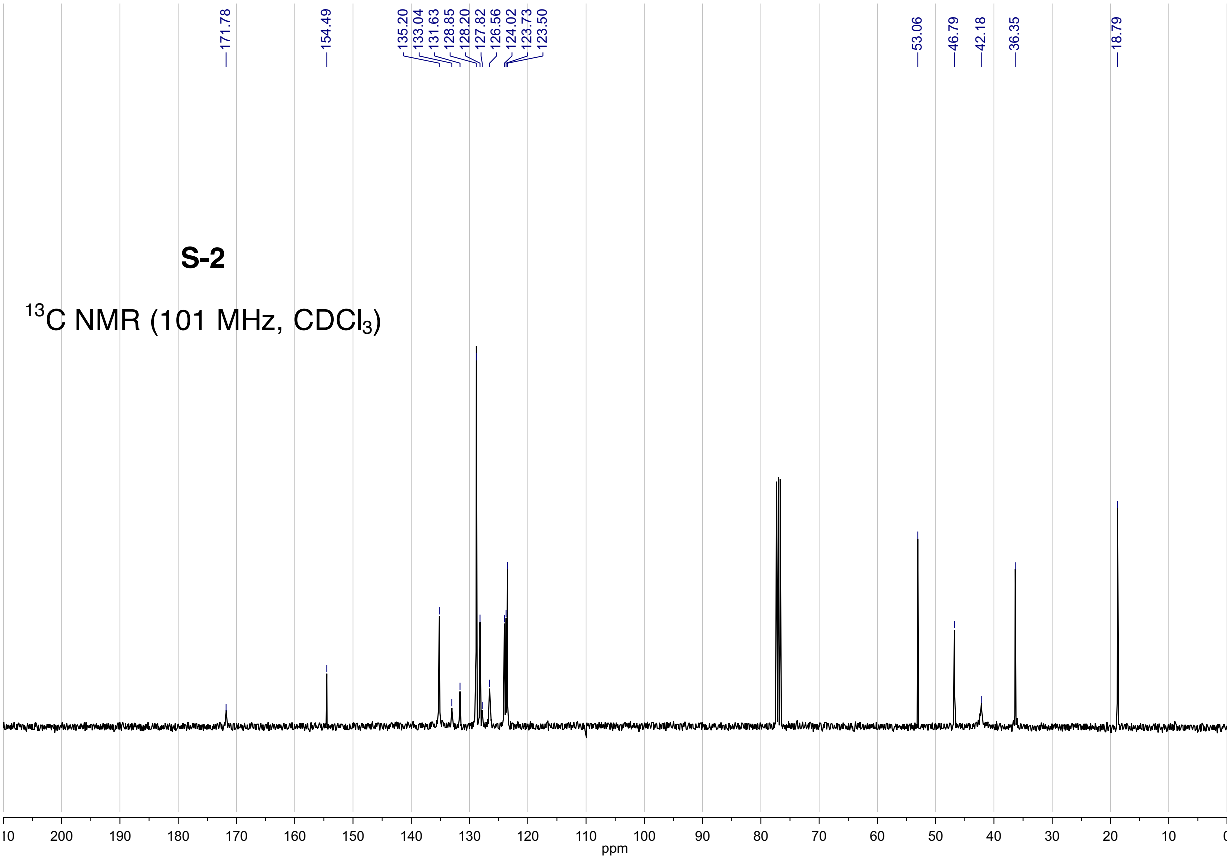
171.78
154.49
135.20 133.04 131.63 128.85 128.20 127.82 126.56 124.02 123.73 123.50
53.06 46.79 42.18 36.35 18.79
S-2
^{13}C NMR (101 MHz, $CDCl_3$)
ppm

Methyl 4-acryloyl-3,4-dihydroquinoxaline-1(2*H*)-carboxylate (2)

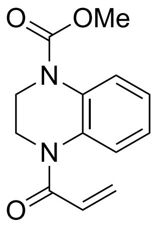

$NaIO_4$ (81 mg, 0.38 mmol) was added to a solution of methyl 4-(2-(phenylselanyl)propanoyl)-3,4-dihydroquinoxaline-1(2*H*)-carboxylate **S-2** (77 mg, 0.19 mmol) in EtOH (1.9 mL), and was stirred at 30 ºC for 18 h. The mixture was then filtered through Celite, washing with EtOAc (5 mL). The solvent was removed under reduced pressure and the crude residue was purified by flash column chromatography (20% grading to 30% EtOAc/pentane) which afforded acrylamide **2** (37 mg, 78%) as a colourless oil.

R_f 0.23 (EtOAc/pentane);

ν_{max} (film)/cm^{-1} 2954, 1708, 1655, 1498, 1409, 1327, 1215, 1056, 764;

^{1}H NMR (400 MHz, $CDCl_3$) δ 7.95–7.86 (m, 1 H), 7.25–7.18 (m, 1 H), 7.11–7.05 (m, 2 H), 6.61 (dd, *J* = 16.8, 10.1 Hz, 1 H), 6.48 (dd, *J* = 16.8, 2.1 Hz, 1 H), 5.74 (dd, *J* = 10.1, 2.1 Hz, 1 H), 4.05–4.00 (m, 2 H), 3.94–3.89 (m, 2 H), 3.83 (s, 3 H);

^{13}C NMR (101 MHz, $CDCl_3$) δ 164.1, 154.6, 132.7, 131.4, 128.8, 128.6, 126.1, 124.6, 123.7, 53.2, 46.8, 42.6 (1 signal missing due to overlap);

HRMS (ESI$^+$) *m/z* Calculated for $C_{13}H_{15}N_2O_3^+$ [M+H]$^+$ 247.1083; Found 247.1091 (Δ +3.2 ppm).

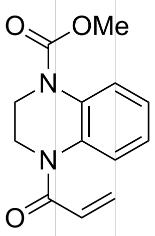

2

^{1}H NMR (400 MHz, $CDCl_3$)

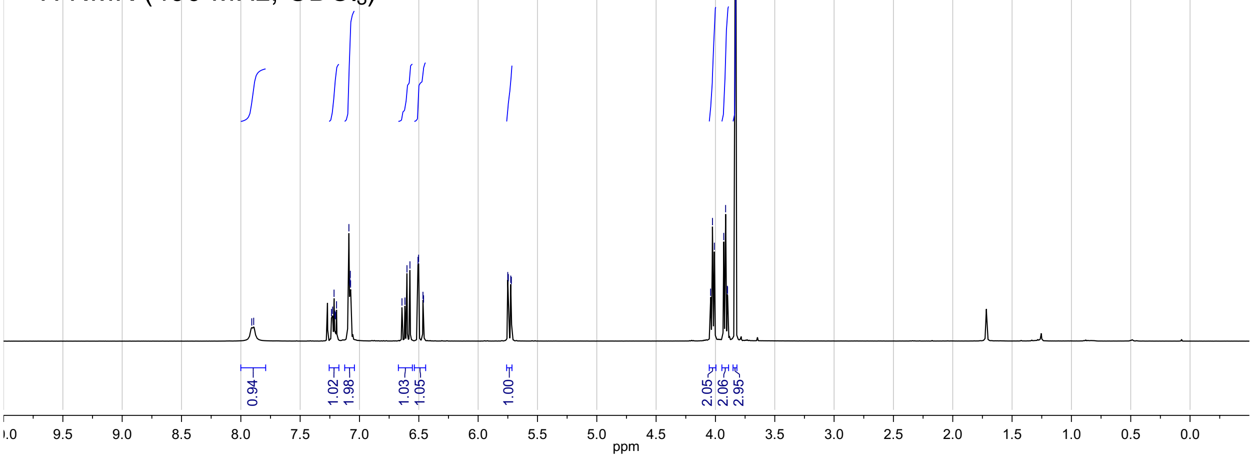

2

^{13}C NMR (101 MHz, $CDCl_3$)

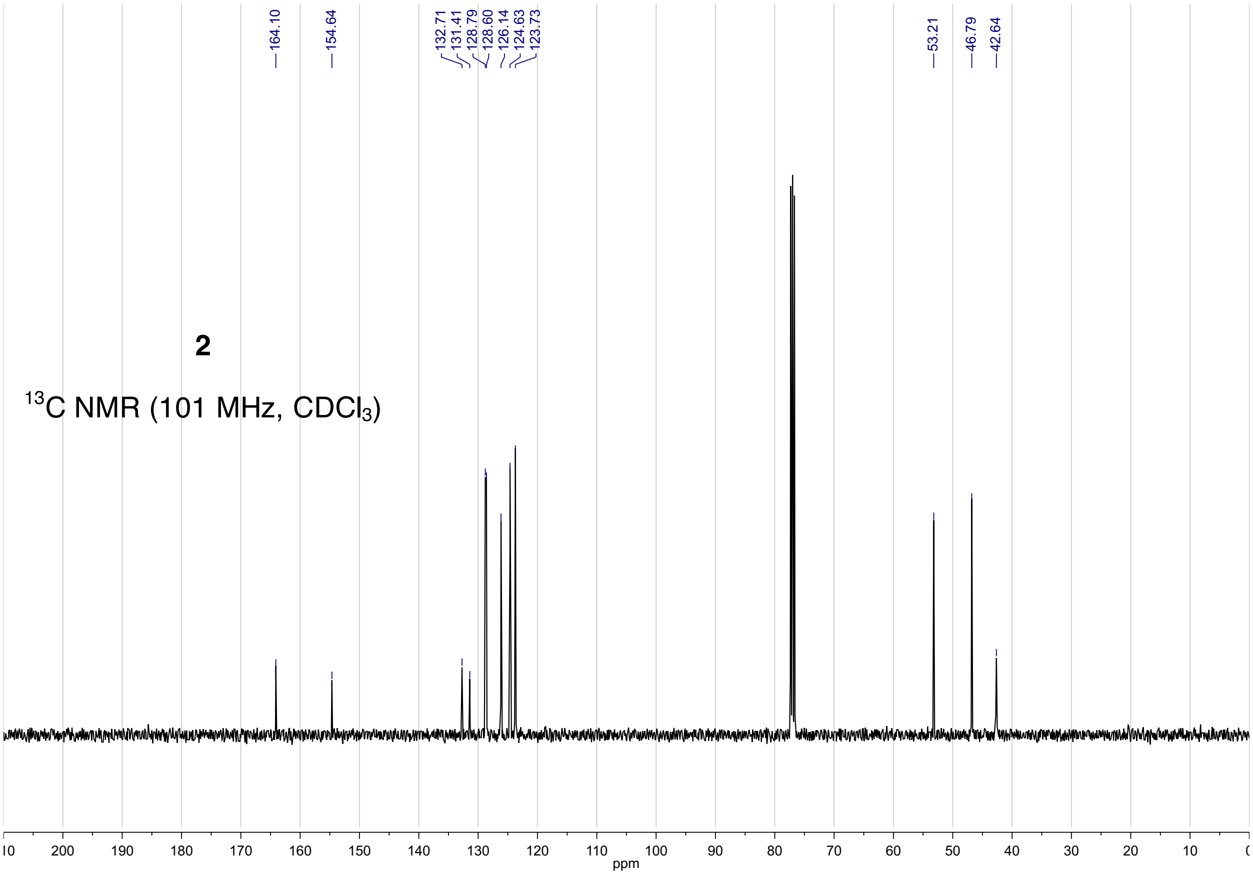

4-(2-(Phenylselanyl)propanoyl)-*N*-(prop-2-yn-1-yl)-3,4-dihydroquinoxaline-1(2*H*)-carboxamide (S-3)

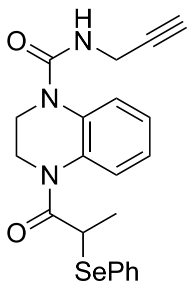

Triphosgene (143 mg, 0.53 mmol) was added to a solution of 1-(3,4-dihydroquinoxalin-1(2*H*)-yl)-2-(phenylselanyl)propan-1-one **S-A** (230 mg, 0.67 mmol) and NEt_3 (140 μL, 1.01 mmol) in CH_2Cl_2 (6.7 mL) at 0 ºC. After 6 h at this temperature sat. aq. $NaHCO_3$ (25 mL) was added, and further CH_2Cl_2 (25 mL) was added. The phases were separated and the organic layer was collected. The aqueous layer was extracted with further CH_2Cl_2 (2 × 25 mL). The organic layers were combined, dried over Na_2SO_4, filtered, and the solvent was removed under reduced pressure, which afforded 4-(2-(phenylselanyl)propanoyl)-3,4-dihydroquinoxaline-1(2H)-carbonyl chloride as a colourless oil, which was used in the next step without further purification.

A solution of 4-(2-(phenylselanyl)propanoyl)-3,4-dihydroquinoxaline-1(2H)-carbonyl chloride (99 mg, 0.24 mmol) in DMF (1.2 mL) was added dropwise to a solution of propargylamine (31 μL, 0.48 mmol), NEt_3 (37 μL, 0.26 mmol) and DMAP (5.9 mg, 48 μmol) in DMF (1.2 mL) at rt. After 24 h, sat. aq. Na-HCO_3 (20 mL) was added and the resulting mixture was extracted with CH_2Cl_2 (3 × 20 mL). The organic layers were combined, dried over Na_2SO_4, filtered and the solvent was removed under reduced pressure. The resulting crude material was purified by flash column chromatography (10% grading to 40% EtOAc/pentane), which afforded propargylamide **S-3** (73 mg, 71%) as a colourless oil.

R_f 0.38 (50% EtOAc/pentane);

ν_{max} (film)/cm^{-1} 3301, 2924, 1644, 1494, 1311, 1215, 728;

^{1}H NMR (400 MHz, $CDCl_3$) δ 7.62–7.18 (m, 8 H), 7.14–7.08 (m, 1 H), 5.32 (s, 1 H), 4.46–4.14 (m, 2 H), 4.12–3.82 (m, 3 H), 3.79–3.21 (m, 2 H), 2.23–2.03 (m ,1 H), 1.63 (d, *J* = 6.8 Hz, 3 H);

^{13}C NMR (101 MHz, $CDCl_3$) δ 171.6, 155.2, 135.6 (2 × C_{Ar}), 133.6, 129.0 (2 × C_{Ar}), 128.6, 127.2, 126.9, 125.3, 124.7, 123.7, 80.1, 71.5, 45.6, 44.6, 41.2, 36.0, 30.6, 18.7;

This compound appeared as a mixture of rotamers in the NMR spectra.

HRMS (ESI$^+$) *m/z* Calculated for $C_{21}H_{22}N_3O_2{}^{80}Se^+$ $[M+H]^+$ 428.0877; Found 428.0873 (Δ –0.9 ppm).

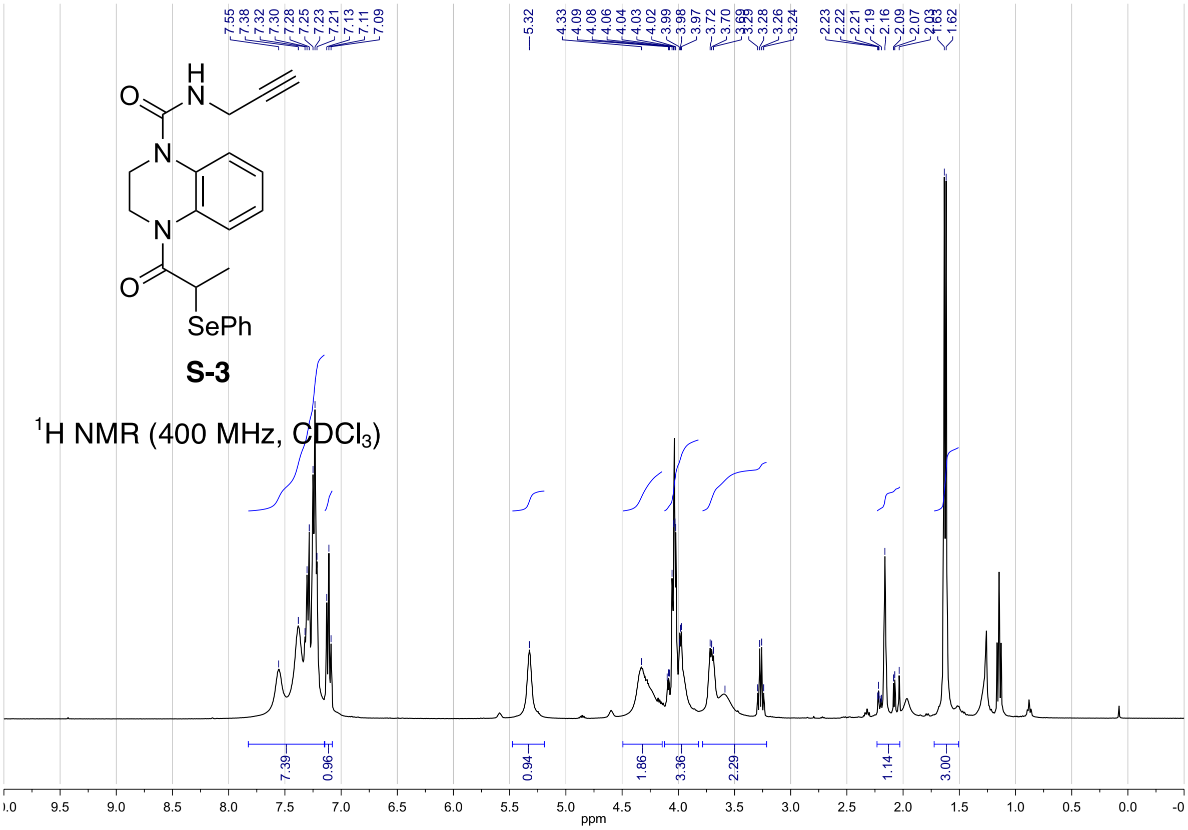
S-3
^{1}H NMR (400 MHz, $CDCl_3$)
ppm

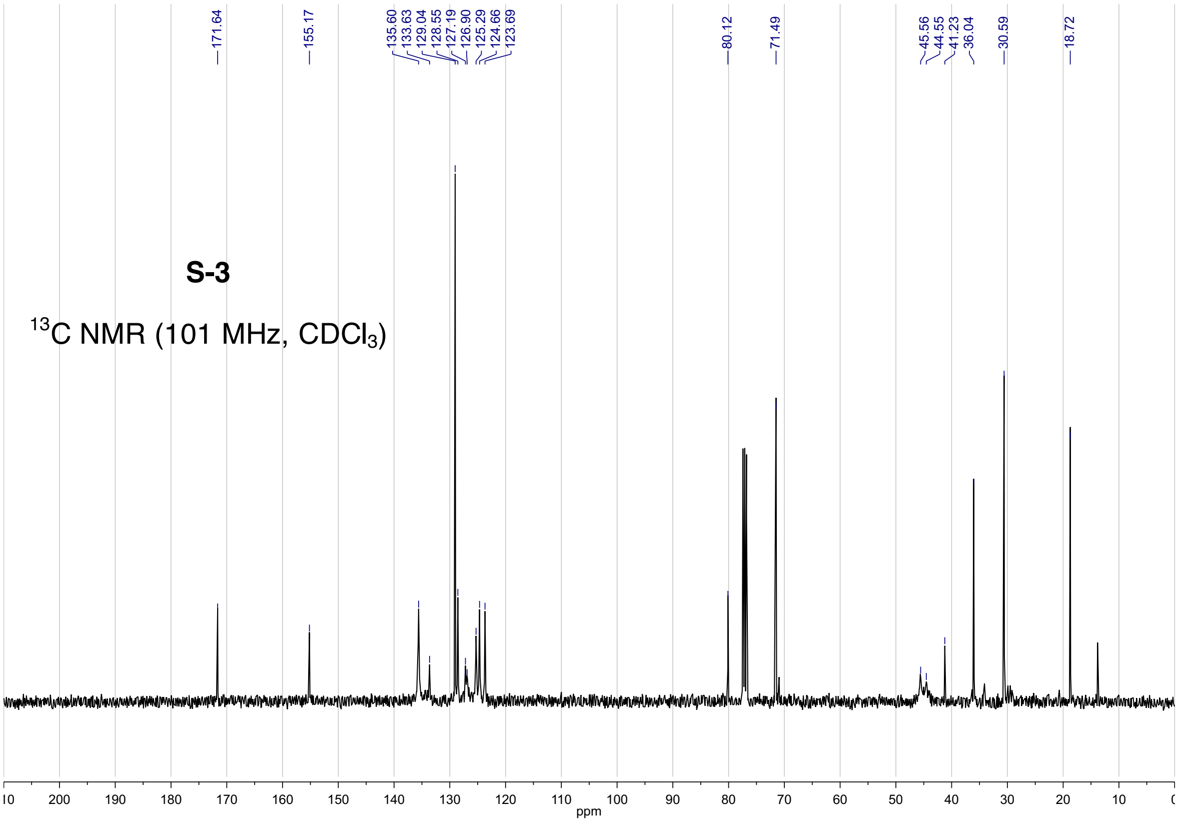
S-3
^{13}C NMR (101 MHz, $CDCl_3$)
ppm

4-Acryloyl-*N*-(prop-2-yn-1-yl)-3,4-dihydroquinoxaline-1(2*H*)-carboxamide (3)

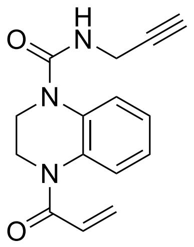

$NaIO_4$ (73 mg, 0.34 mmol) was added to a solution of 4-(2-(phenylselanyl)propanoyl)-*N*-(prop-2-yn-1-yl)-3,4-dihydroquinoxaline-1(2*H*)-carboxamide **S-3** (73 mg, 0.17 mmol) in EtOH (1.7 mL), and was stirred at 30 ºC for 18 h. The mixture was then filtered through Celite, washing with EtOAc (5 mL). The solvent was removed under reduced pressure and the crude residue was purified by flash column chromatography (25% grading to 75% EtOAc/pentane) which afforded acrylamide **3** (29 mg, 64%) as a colourless oil.

R_f 0.24 (50% EtOAc/pentane);

ν_{max} (film)/cm^{-1} 3291, 2942, 1646, 1615, 1496, 1412, 1318, 1220, 759;

^{1}H NMR (400 MHz, $CDCl_3$) δ 7.52 (d, *J* = 8.6 Hz, 1 H), 7.26–7.19 (m, 2 H), 7.18–7.11 (m, 1 H), 6.60 (dd, *J* = 16.8, 10.0 Hz, 1 H) 6.48 (dd, *J* = 16.9, 2.0 Hz, 1 H), 5.75 (dd, *J* = 10.0, 2.1 Hz, 1 H), 5.30 (t, *J* = 5.4 Hz, 1 H), 4.08 (dd, *J* = 5.3, 2.5 Hz, 2 H), 4.03 (t, *J* = 6.5 Hz, 2 H), 3.92 (t, *J* = 6.5 Hz, 2 H), 2.25 (t, *J* = 2.6 Hz, 1 H);

^{13}C NMR (101 MHz, $CDCl_3$) δ 164.7, 154.8, 133.5, 133.1, 128.9, 128.7, 126.2, 125.5, 124.8, 123.4, 80.0, 71.5, 45.2, 45.0, 30.6;

HRMS (ESI$^+$) *m/z* Calculated for $C_{15}H_{16}N_3O_2^+$ [M+H]$^+$ 270.1243; Found 270.1247 (Δ +1.5 ppm).

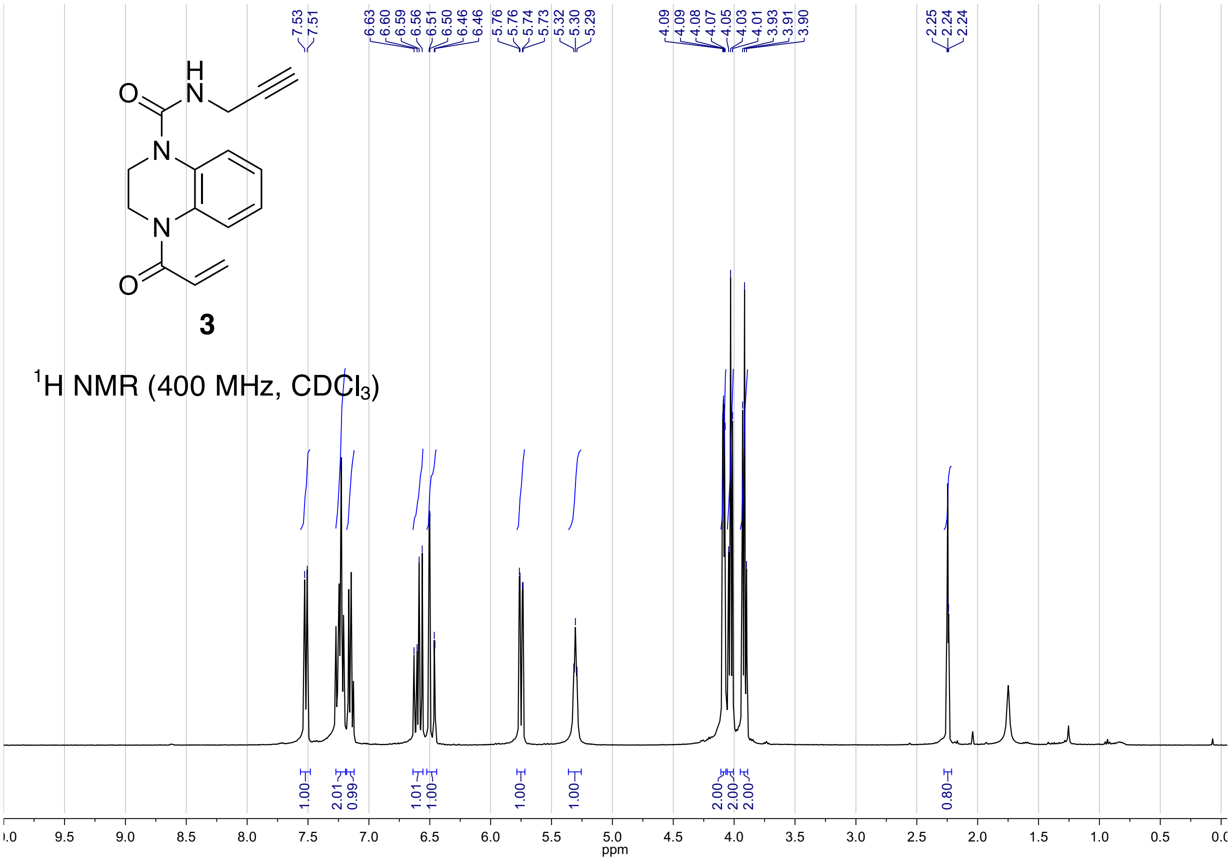

3

^{1}H NMR (400 MHz, $CDCl_3$)

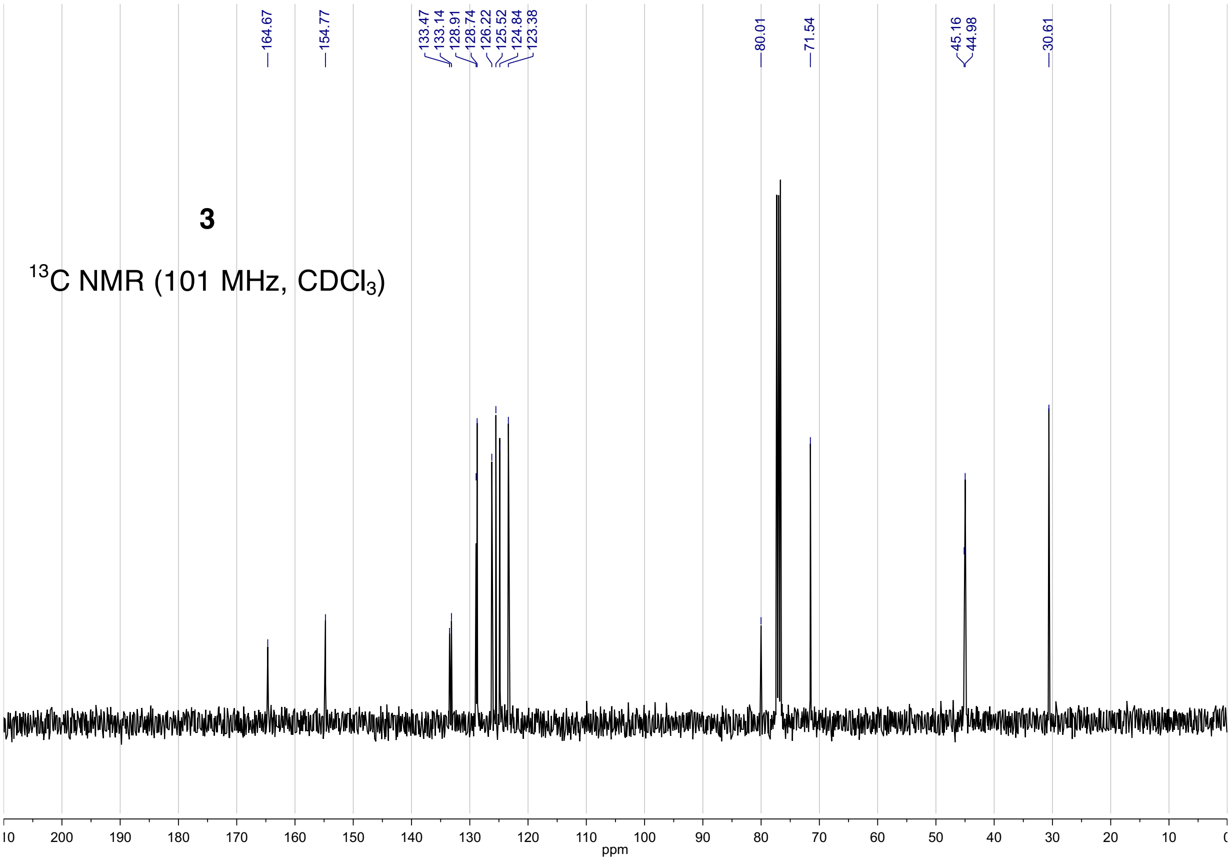

3

^{13}C NMR (101 MHz, $CDCl_3$)

tert-Butyl 4-acryloylpiperazine-1-carboxylate (4)

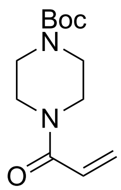

Acrylic acid (6.8 µL, 0.10 mmol) and *tert*-butyl piperazine-1-carboxylate (19 mg, 0.10 mmol) were added to a solution of PS-IIDQ (133 mg, 0.20 mmol, 1.5 mmolg^{-1}) in MeCN (3.0 mL), and the reaction was shook for 24 h at rt. The mixture was then filtered through Celite, washed with CH_2Cl_2:MeOH (1:1, 10 mL), and the filtrate was concentrated under reduced pressure. CH_2Cl_2 (5 mL) and sat. aq. $NaHCO_3$ (5 mL) were added and the phases were separated. The aqueous layer was extracted with CH_2Cl_2 (3 × 5 mL), and the combined organic layers were dried over $MgSO_4$. The solvent was removed under reduced pressure and the crude residue was purified by ion exchange chromatography on an acidic resin, eluting with MeOH, which afforded acrylamide **4** (13 mg, 54%) as a colourless oil.

^{1}H NMR (400 MHz, $CDCl_3$) δ 6.59 (dd, *J* = 17.0, 10.5 Hz, 1 H), 6.34 (dd, *J* = 17.0, 2.0 Hz, 1 H), 5.76 (dd, *J* = 10.5, 2.0 Hz, 1 H), 3.72–3.66 (m, 2 H), 3.61–3.55 (m, 2 H), 3.51–3.46 (m, 4 H), 1.51 (s, 9 H);

HRMS (ESI$^+$) *m/z* Calculated for $C_{12}H_{20}N_2O_3Na^+$ $[M+Na]^+$ 263.1372; Found 263.1360 (Δ –4.6 ppm).

The ^{1}H NMR spectroscopy and mass spectrometry data match those previously described in the literature.[1]

tert-Butyl 6-nitro-3,4-dihydroquinoxaline-1(2_H_)-carboxylate (S-5)

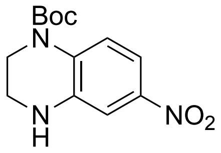

A di-*tert*-butyl dicarbonate solution (6.09 mL, 1.0 M in THF, 6.09 mmol) and NEt_3 (1.73 mL, 13.00 mmol) were added to a solution of 6-nitro-1,2,3,4-tetrahydroquinoxaline (800 mg, 4.06 mmol) and DMAP (99 mg, 0.81 mmol) in CH_2Cl_2 (20 mL). The resulting mixture was heated to 35 ºC for 1 h. The reaction mixture was allowed to cool to rt and was washed with 1.0 M aq. HCl (20 mL), then sat. aq. $NaHCO_3$ (20 mL), and then brine (20 mL). The organic layer was dried over Na_2SO_4, filtered, and concentrated under reduced pressure. The crude residue was purified by flash column chromatography (5% grading to 50% acetone/pentane) to give *tert*-butyl 6-nitro-3,4-dihydroquinoxaline-1(2*H*)-carboxylate **S-5** (985 mg, 87%) as an orange solid.

m.p. = 122–124 ºC ($CHCl_3$)

R_f 0.25 (10% acetone/pentane)

ν_{max} (film)/cm^{-1} 3391, 2978, 1690, 1518, 1332, 1146, 743;

^{1}H NMR (400 MHz, $CDCl_3$) δ 7.68 (d, *J* = 9.0 Hz, 1 H, BocN$C_{Ar}C_{Ar}H$), 7.49 (dd, *J* = 9.0, 2.6 Hz, 1 H, BocN$C_{Ar}C_{Ar}HC_{Ar}H$), 7.42 (d, *J* = 2.6 Hz, 1 H, NH$C_{Ar}C_{Ar}H$), 4.43 (s, 1 H, NH), 3.83–3.78 (m, 2 H, CH_2), 3.48–3.42 (m, 2 H, CH_2), 1.54 (s, 9 H, $C(CH_3)_3$).

^{13}C NMR (101 MHz, $CDCl_3$) δ 152.5, 144.1, 136.9, 130.2, 124.0, 111.8, 108.9, 82.2, 41.4, 41.3, 28.2 (3 × CH_3).

HRMS (ESI$^+$) *m/z* Calculated for $C_{13}H_{18}N_3O_4^+$ $[M+H]^+$ 280.1297, Found 280.1301 (Δ +1.4 ppm).

The regioselectivity of the Boc protection was determined by nOe analysis.

S-5

^1H NMR (400 MHz, $CDCl_3$)

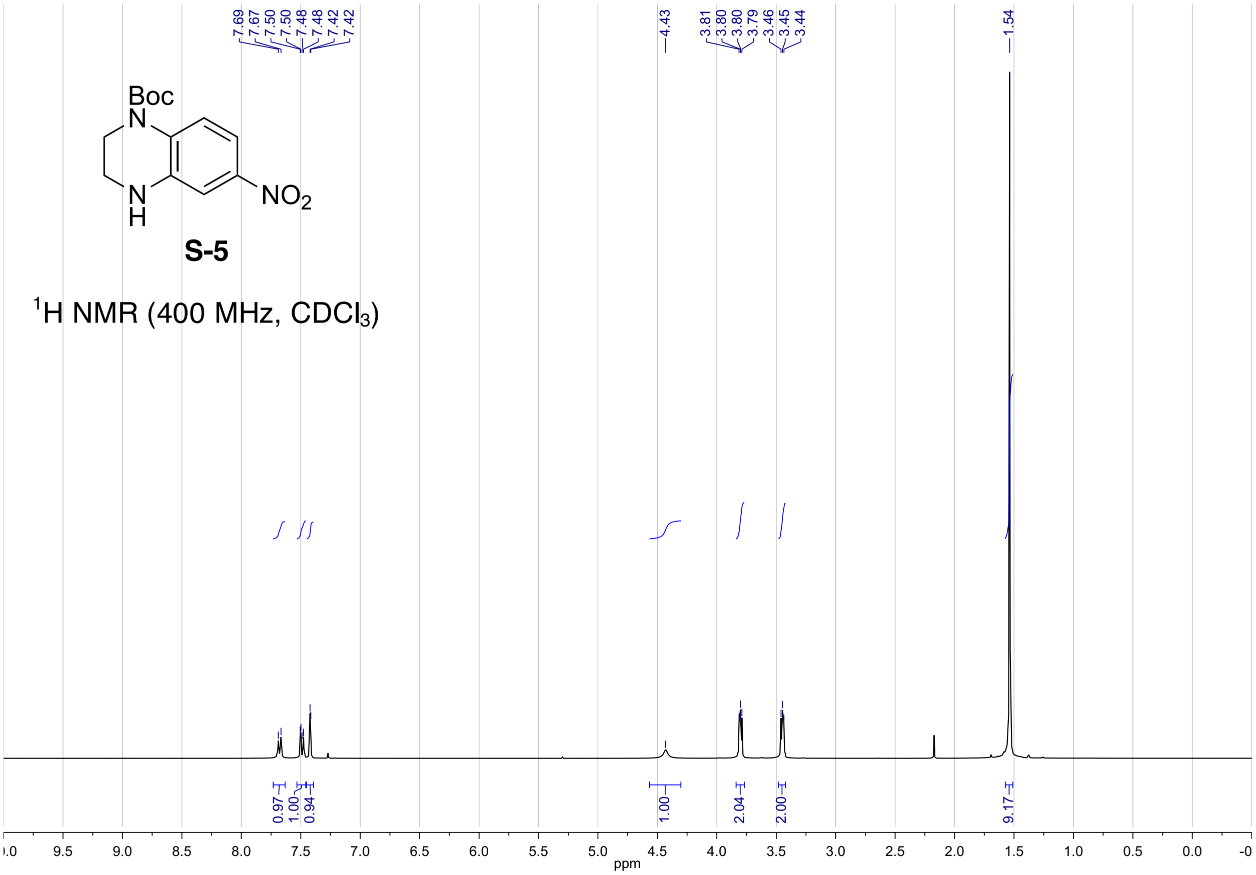

S-5

^{13}C NMR (101 MHz, $CDCl_3$)

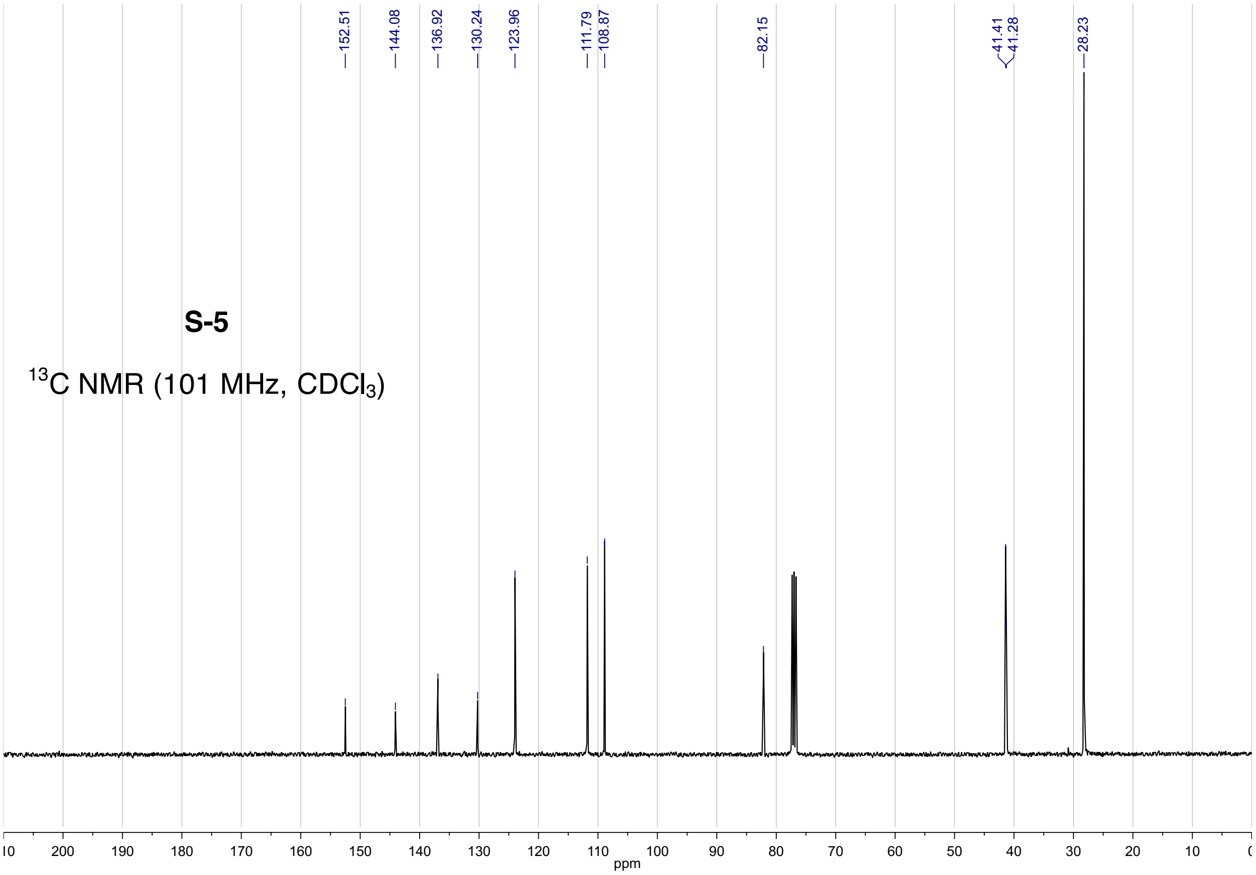

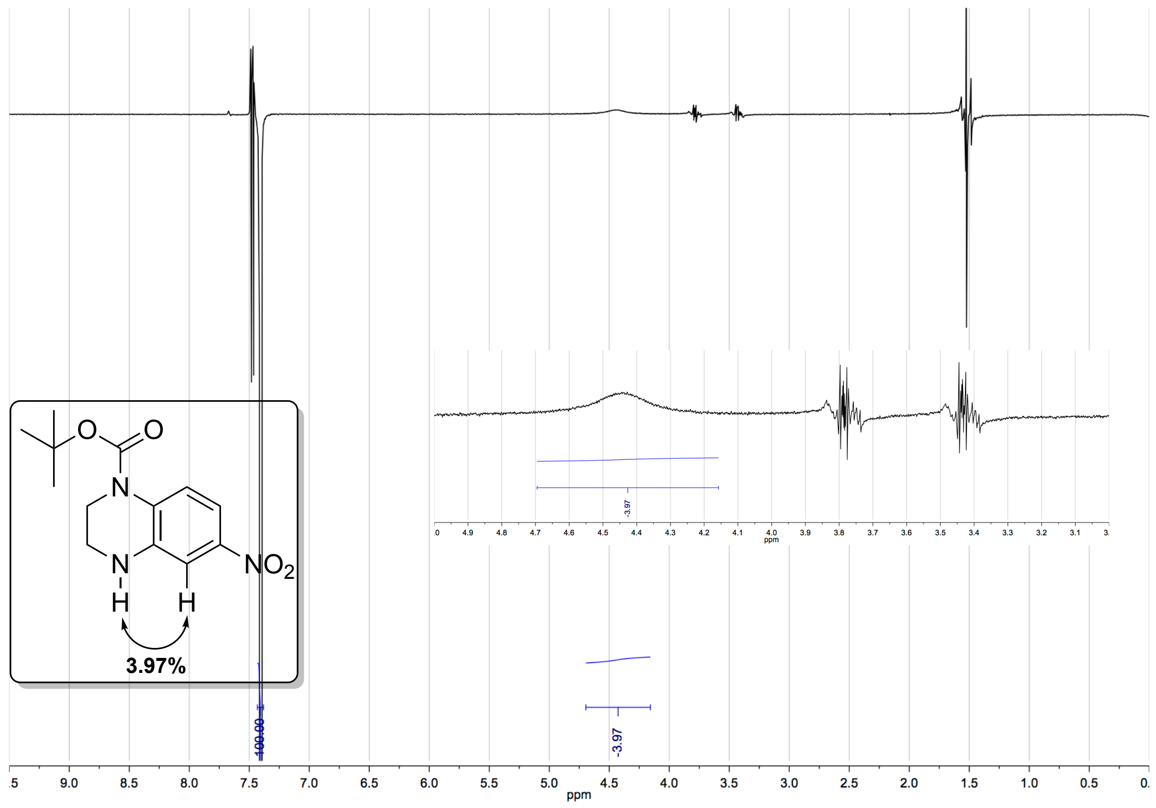

NO2
N
H
H
3.97%
-3.97
-100.00
ppm

tert-Butyl 4-acryloyl-6-nitro-3,4-dihydroquinoxaline-1(2*H*)-carboxylate (5)

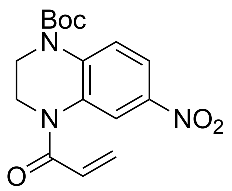

Modifying our previously reported procedure,[2] a solution of acryloyl chloride in CH_2Cl_2 (400 µL, 3.6 M, 1.44 mmol) was added to a solution of *tert*-butyl 6-nitro-3,4-dihydroquinoxaline-1(2*H*)-carboxylate **S-5** (200 mg, 0.72 mmol) in $CHCl_3$ (2.1 mL). After 5 min, Amberlyst A26(OH) resin (190 mg) was added, and the resulting suspension was heated to 30 ºC for 18 h. The reaction mixture was then filtered through Celite washing with CH_2Cl_2 (5 mL) and concentrated under reduced pressure. The crude residue was purified by flash column chromatography (10% grading to 50% EtOAc/pentane), which afforded acrylamide **5** (233 mg, 98%) as a yellow solid.

m.p. = 53–55 ºC (CH_2Cl_2)

R_f 0.30 (20% EtOAc/pentane)

ν_{max} (film)/cm^{-1} 2983, 1711, 1659, 1517, 1497, 1303, 1144, 913, 727;

^{1}H NMR (400 MHz, $CDCl_3$) δ 8.24 (d, *J* = 9.2 Hz, 1 H), 8.07–8.00 (m, 2 H), 6.63–6.54 (m, 2 H), 5.86 (dd, *J* = 8.0, 4.0 Hz, 1 H), 4.05–3.99 (m, 2 H), 3.94–3.89 (m, 2 H), 1.56 (s, 9 H).

^{13}C NMR (101 MHz, $CDCl_3$) δ 163.8, 152.5, 142.1, 138.5, 130.6, 130.4, 127.6, 123.1, 121.1, 119.7, 83.3, 47.3, 41.5, 28.1 (3 × CH_3).

HRMS (ESI$^+$) *m/z* Calculated for $C_{16}H_{20}N_3O_5^+$ [M+H]$^+$ 334.1403, Found 334.1409 (Δ +1.8 ppm).

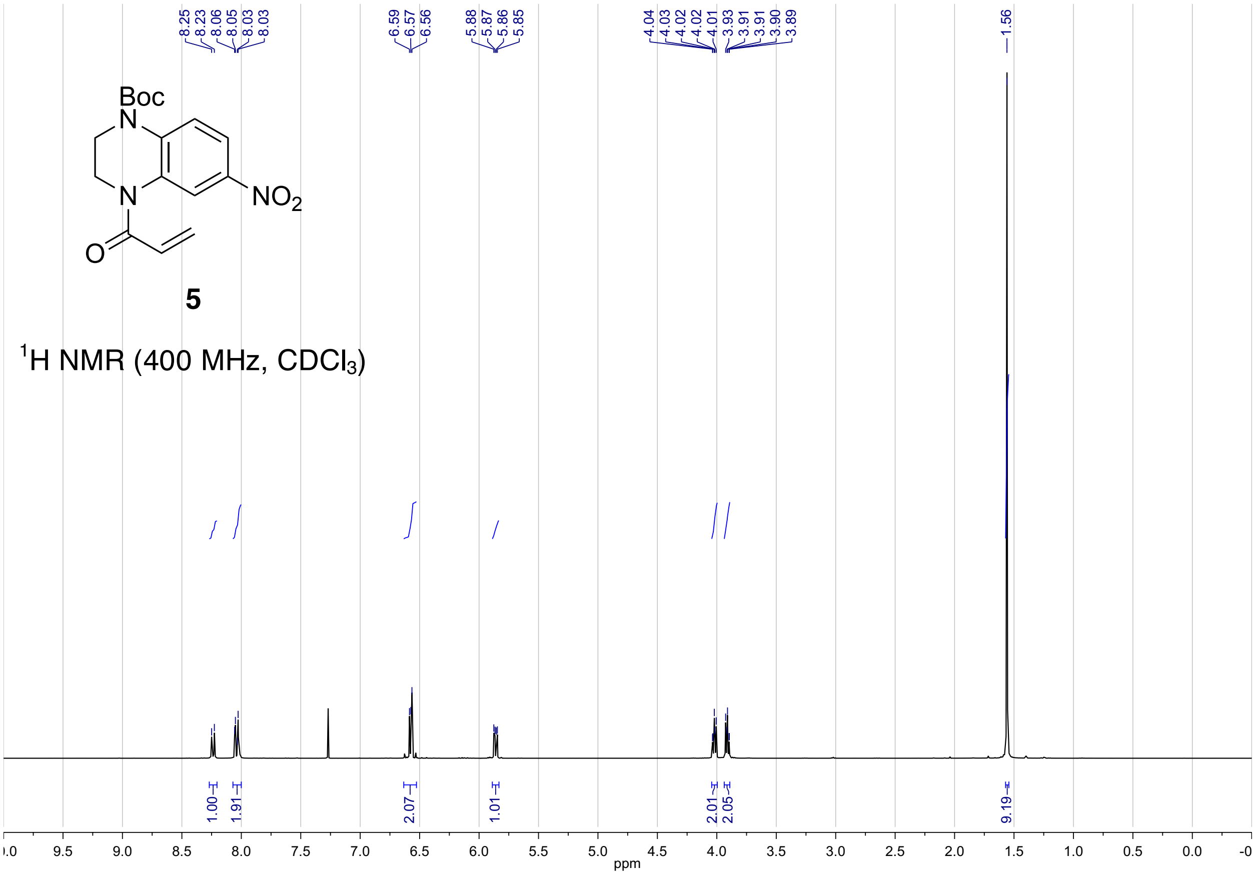

Boc
N
N
NO_2
O
5
^{1}H NMR (400 MHz, $CDCl_3$)
8.25
8.23
8.06
8.05
8.03
8.03
6.59
6.57
6.56
5.88
5.87
5.86
5.85
4.04
4.03
4.02
4.02
4.01
3.93
3.91
3.91
3.90
3.89
1.56
1.00
1.91
2.07
1.01
2.01
2.05
9.19
ppm

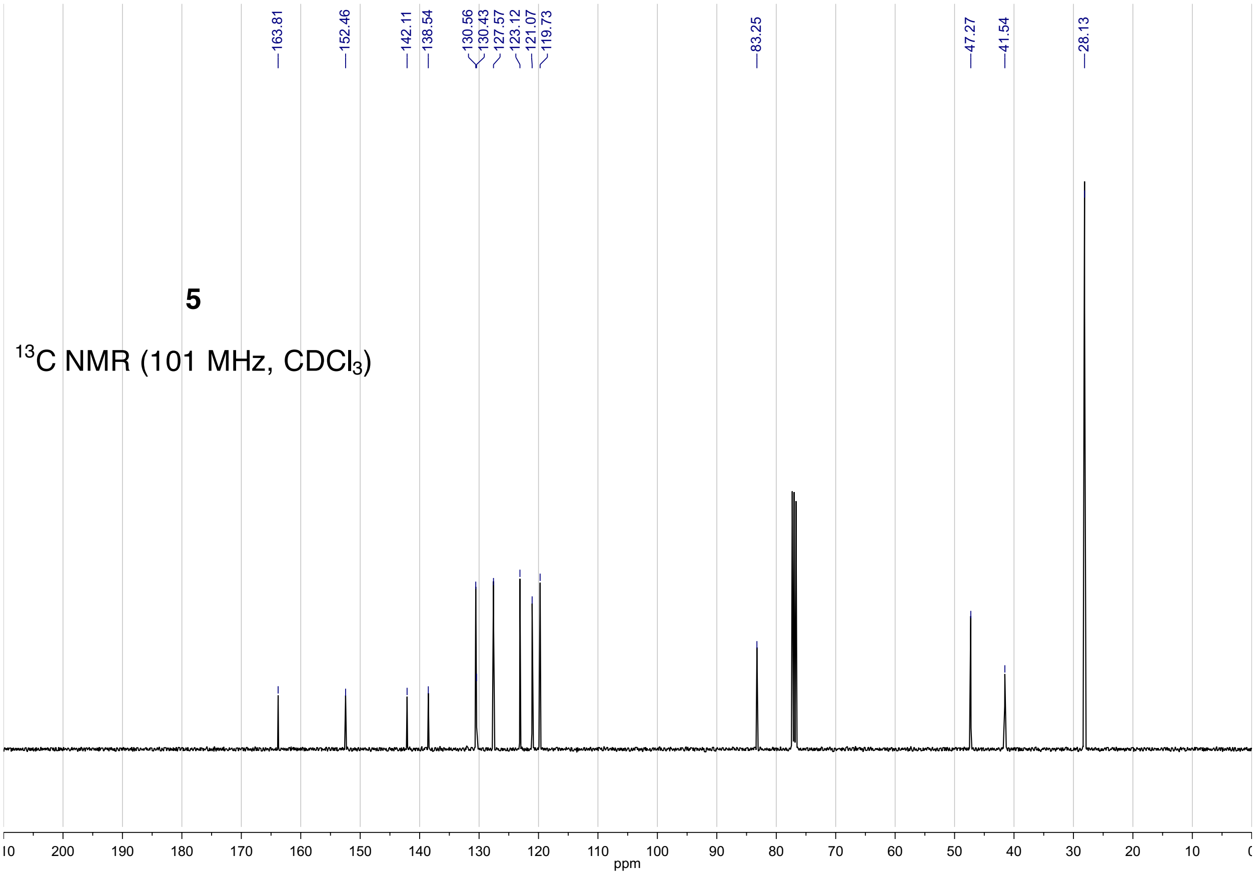

163.81
152.46
142.11
138.54
130.56
130.43
127.57
123.12
121.07
119.73
83.25
47.27
41.54
28.13
5
^{13}C NMR (101 MHz, $CDCl_3$)
ppm

C. Library design

Acrylamide fragments were synthesized from amine precursors. In brief, 14,000 primary and secondary amines were selected from Enamine's commercial collection with molecular weight < 250 Da, from which 235 unique Bemis-Murcko frameworks were generated.[3] For 100 of the frameworks, the lowest-molecular weight example was purchased. The corresponding acrylamides were then synthesized in a one step acrylation reaction (see *Efficient and Facile Synthesis of Acrylamide Libraries for Protein-Guided Tethering* for full synthetic details).[2] The rest of the library consisted of commercially available electrophilic fragments, with warheads including epoxide, chloroacetamide, vinylsulfone, chloropyridine and cyanamide (Supplementary Fig. S2a). The physiochemical properties of the final library were calculated using DataWarrior[4] and found to broadly comply with the 'Rule of 3' guidelines[5], with the recommended relaxed restriction on number of H-bond acceptors[6] (Supplementary Fig. S2b).

D. Rate determination by qIT

Reaction-plate setup: Each well of a 96-well PCR plate was charged with 60–100 µL of target thiol (GSH or protein, 10 µM) and 4% w/v TCEP-agarose beads in 100 mM phosphate buffer (pH 7.5 or 8) and cooled to 4 ˚C. Reactions were started by addition of an equal volume of pre-cooled electrophile solution (1 mM, 2% DMSO) in 0.1 M phosphate buffer (final concentration: thiol = 5 µM, electrophile = 0.5 mM, 2% w/v TCEP-agarose beads), with a minimum of 4 wells per plate used as DMSO/thiol only controls. After mixing, TCEP-agarose was pelleted by centrifugation (1,300 rpm, 1 min) and the plate incubated at 4 ˚C. *CPM Quench*: At a series of time points (typically around 0.25, 0.5, 1, 2, 6, 24, 48, 72 and 120 hours after reaction initiation), 3 µL aliquots of each reaction were quenched into a 384-well fluorescence plate (Corning – black, NBS) where each well was charged with 27 µL of CPM solution (1.39 µM) in 100 mM phosphate buffer (pH 7.5) (final concentration: thiol = 0.5 µM, CPM = 1.25 µM). Quenching was typically performed in duplicate and fluorescence plates were incubated at room temperature for 60 minutes before fluorescence intensity (excitation/emission: 384/470 nm) was measured on an EnVision™ plate reader. *Analysis*: All analysis was performed using Prism 6.0 software (GraphPad). For each time point, the fluorescence of each reaction was normalized to the average of the DMSO/thiol only controls (normalized fluorescence = reaction fluorescence/control fluorescence). The normalized fluorescence for each reaction was plotted against time. A one phase exponential decay was fitted to each plot (Constraints: Y(0) > 0.5; 0 < plateau < 0.3; *k* > 0), yielding a pseudo first-order rate constant.

E. Z' Factor determination

Thiol solutions (200µL, 5 µM, positives = Cdk2(WT) and glutathione; negatives = Cdk2(C177A) and glutamate) were incubated with TCEP-agarose (2% v/v). After 28, 68 and 140 hours, fluorogenic quantifications were performed for each thiol (n = 72), using the CPM quench procedure detailed under Methods - Rate determination by qIT. Z' factors were calculated using the formula $Z' = 1 - (3(\sigma_p + \sigma_n)/|\mu_p - \mu_n|)$, where σ_p = standard deviation of positives, σ_n = standard deviation of negatives, μ_p = mean of positives, μ_n = mean of negatives.

F. Protein expression

Plasmids expressing His_6-Cdk2 constructs were used for screening and GST-Cdk2 for crystallography, kinase assays and TdCD. Cdk2 mutations were introduced by PCR (see section F for primers) and cloned into pRSETA for expression of His_6-tagged protein. pGEX6P1 Cdk2 (552), a gift from Jonathon Pines (Addgene plasmid # 61845) was used to express GST-Cdk2(WT) and other mutants were constructed by introducing appropriate fragments of the pRSETA plasmids into pGEX6P1 Cdk2. All constructs were verified by sequencing. pGEX6P1 cyclin A2 (amino acids 173-432) was generated by PCR and restriction digestion. GST-CAK1 was constructed by PCR amplification of *S.cerevisiae* genomic DNA (see below for primers) and the product cloned into pKEG-KG. All protein expression was performed using an adapted autoinduction protocol:[7] Plasmids were transformed into E. coli Tuner(DE3) and cultures grown in autoinduction media at 37˚C until OD_{600} = 0.9. The cultures were then incubated at 18˚C for a further 40 hours before harvesting by centrifugation (10 min, 6000 g).

G. Primers

Cdk2 F 5' GATCGGATCCATGGAGAACTTCCAAAAGGTGG
Cdk2 R 5' GATCGAATTCTCAGAGTCGAAGATGGGGTACTGGC
F80C F 5' CTCTACCTGGTTTGCGAATTTCTGCAC
F80C R 5' GTGCAGAAATTCGCAAACCAGGTAGAG
H71C F 5' GCTGGATGTCATATGCACAGAAAATAAACTC
H71C R 5' GAGTTTATTTTCTGTGCATATGACATCCAGC
R122C F 5' CTTTCTGCCATTCTCATTGCGTCCTCCACCGAG
R122C R 5' CTCGGTGGAGGACGCAATGAGAATGGCAGAAAG
D145N F 5' ccatcaagcttgcaaactttggactagc
D145N R 5' gctagtccaaagtttgcaagcttgatgg
C177A F 5' GAAATCCTCCTGGGCGCCAAATATTATTCC
C177A R 5' GGAATAATATTTGGCGCCCAGGAGGATTTC

S181C F 5' GCCAAATATTATTGCACTGCAGTGGACATCTGG
S181C R 5' CCAGATGTCCACTGCAGTGCAATAATATTTGGC
T182C F 5' CCAAATATTATTCCTGCGCTGTCGACATCTGGAGCC
T182C R 5' GGCTCCAGATGTCGACAGCGCAGGAATAATATTTGG
N272C F 5' GCTGCACTACGACCCTTGCAAAAGGATTTCGGCC
N272C R 5' GGCCGAAATCCTTTTGCAAGGGTCGTAGTGCAGC
S276C F 5' GACCCTAACAAGCGCATATGCGCCAAGGCAGCCCTG
S276C R 5' CAGGGCTGCCTTGGCGCATATGCGCTTGTTAGGGTC
K278C F 5' GCGGATTTCGGCATGCGCAGCCCTGGCTCAC
K278C R 5' GTGAGCCAGGGCTGCGCATGCCGAAATCCGC

Hs Cyclin A2 F 5' GGATCCAATGAAGTACCAGACTACCATGAGGATATTC
Hs Cyclin A2 R 5' GAATTCACAGATTTAGTGTCTCTGGTGGGTTG

Sc CAK1 F 5' GGATCCATGAAACTGGATAGTATAGACATTAC
Sc CAK1 R 5' GAATTCttatgggcttttctaattcttgcaag

H. Protein purification

GST-Cdk2 and GST-cyclin A2: Harvested cells were resuspended in 50 mM HEPES buffer (pH7.5) containing 150 mM NaCl, 10 mM $MgCl_2$, 2 mM dithiothreitol (DTT), 0.5 mg mL^{-1} lysozyme, and 0.01% Triton X-100 at 4 ˚C and incubated for 30 minutes. After sonication and centrifugation (40 min, 16000 g), the supernatant was incubated for 1 h with glutathione-Sepharose beads, washed with lysis buffer and the desired protein eluted with 10 mM GSH (100 mM Tris, pH 8). After incubation the desired protein was cleaved from the GST tag with PreScission protease (100:1) at 4˚C for 4 hours, the cleaved GST tag was removed with glutathione-Sepharose beads. Cdk2 was concentrated and then loaded onto a Superdex 75 (16/60) column, eluting with 100 mM phosphate buffer containing 50 mM DTT. Purified Cdk2 was concentrated to 10 mg mL^{-1} and stored at – 80 ˚C. Cyclin A2 was purified by glutathione-Sepharose affinity as described above with the following amendments: 100 mM $MgCl_2$ was added after GST cleavage to minimise protein aggregation and gel filtration was performed with 50 mM HEPES buffer (pH 7.5) containing 150 mM NaCl, 100 mM $MgCl_2$, 2 mM DTT. GST-CAK1 was also purified using the glutathione-Sepharose affinity workflow, however no protease or gel filtration steps were performed and the enzyme was used crude.
His_6-Cdk2: Cell lysis was performed as above with the addition of 30 mM imidiazole to the lysis buffer. Following sonication the supernatant was incubated for 1 hour with nickel sulfate charged NTA agarose beads, washed with lysis buffer supplemented with 50 mM imidiazole and then His_6-Cdk2 eluted with lysis buffer containing 500 mM imidiazole. After dialysis against 100 mM phosphate buffer (pH 8) containing 1 mM DTT, His_6-Cdk2 was concentrated and loaded onto a Superdex 75 (16/60) column, eluting with 100 mM phosphate buffer (pH 8) without reducing agent.

I. Acrylamide labelling of Cdk2 for crystallography, TdCD, kinase assays and intact-protein mass spectrometry

Cdk2 (10 μM) was reacted with acrylamide ligands at 500 μM in 100 mM phosphate buffer (pH 8) containing 1% DMSO, until labelling reached completion (as monitored by fluorescence quench assay and/or intact protein mass spectrometry). Excess ligand was subsequently removed by serial dilution and concentration (×5) into the appropriate buffer using Amicon Ultra-0.5 centrifugal filter devices (MW cut-off = 10 kDa).

J. Intact-protein mass spectrometry

Protein-mass data were obtained on a Waters micromass LC-TOF mass spectrometer in positive ion mode using electrospray ionization. Samples were chromatographed using an Acquity UPLC protein BEH C4 column (2.1 mm × 50 mm, 2.7 μm) with a flow rate of 30 μL/min. The injection volume was 5 μL of protein solution (5 μM) in 30 mM ammonium bicarbonate buffer containing 0.5% formic acid and 20% MeOH. The gradient used was 100% mobile phase A (0.1% formic acid in water/MeOH (19:1)) ramping linearly to 90% mobile phase B (0.1% formic acid in MeOH) over 5 minutes. The spectra were acquired from 500 to 2500 Da and deconvolution was performed with MassLynx (Waters), using the maximum entropy algorithm, over a mass range of 800 to 1200 Da with a mass step of 0.75.

K. Crystallography

All crystallization was performed using the hanging drop method at 21˚C and crystals were harvested in cryoprotectant: 30% w/v Jeffamine ED-2003, 100 mM HEPES, pH 7.5. *Apo-Cdk2:* Crystallisation was performed by mixing 1 μL of Cdk2(WT) solution (8 mg mL^{-1}, 100 mM phosphate buffer, pH 6.2 containing 1 mM DTT) with 1 μL of reservoir solution (30% w/v Jeffamine ED-2003, 100 mM HEPES, pH range 7.5 – 8.5). Crystals appeared overnight (typical dimensions: 150 μm^3) and diffracted to 1.2 Å. *Microseed preparation*: Microseeds were generated using a glass seed bead kit (Hampton Research) according to the manufacturer's description. In brief, three apo-Cdk2(WT) crystals were pipetted into the seed glass tube, containing 50 μL of reservoir solution (30% w/v Jeffamine ED-2003, 100 mM HEPES, pH 7.5), and vortexed twice for ten seconds. An additional 450 μL of reservoir solution was added to form a high-concentration seed stock. Empirically, it was found that an additional

100-fold dilution gave the optimum seed concentration for subsequent crystallization. *Ligand conjugated Cdk2*: Crystallisation was performed by mixing 1 µL of labelled Cdk2 solution (8 mg mL^{-1}, 100 mM phosphate buffer) with 0.5 µL of reservoir solution (Jeffamine ED-2003 concentration range 18 - 30% w/v, 100 mM HEPES, pH range 7.5 – 8.5) and 0.5 µL diluted apo-Cdk2(WT) microseed stock. For labelled Cdk2(WT), crystals appeared within 2–3 days and grew for another 10–14 days.

L. Molecular dynamics simulation

MD was carried out using the GROMACS package[8]. Covalent ligands were treated as non-standard amino acids. Force field parameters for these were obtained from ATB[9] and by manual comparison of the amino acid to existing amino acids. MD runs were conducted using the GROMOS96 54A7 force field, periodic boundary conditions, SPC water, charge-neutralizing counter ions and a 2 fs timestep. ModLoop[10] was used to model missing residues. An initial energy minimisation was conducted using a steepest descent energy minimization of 5000 steps in a vacuum. This was followed by a constant temperature and volume equilibration for 100 ps, then a constant pressure and temperature equilibration for 100 ps. Production MD was run for 50 ns. Representative structures were found from the trajectory using MDTraj[11].

M. Data collection and structure determination

X-ray diffraction data were collected on the synchrotron beam lines i03 and i04 at Diamond Light Source, Didcot, and processed using XDS[12] and implemented within Xia2[13]. Data collection and refinement statistics are summarized in Supplementary Tables 2-6. Molecular replacement was conducted on all data sets using Phaser[14]/MOLREP[15] against apo-Cdk2 (PDB: 4EK3) and the model was iteratively refined using Phenix.Refine[16] with manual modelling and adjustments carried out in COOT[17]. Descriptions of the ligands and links were generated with JLigand[18] and refined with Refmac[19]. Electron density maps were calculated using FFT[20] and figures were prepared using PyMol (Schrödinger).

N. Cdk2 phosphorylation

Cdk2 (1 mg) was sequentially incubated with aliquots of CAK1 (3 × 0.05 mg) at r.t. for 4 hours at a time in 1.5 mL of a 30 mM Tris/HCl buffer (pH 7.5) containing 10 mM $MgCl_2$, 10 mM ATP and 3 mM DTT. As CAK1 appeared to become rapidly inactivated in the presence of pCdk2, three repetitions were required to drive the reaction to completion. The resulting solution was incubated with 50 µL of glutathione-Sepharose beads and filtered to remove remaining CAK1.

Phosphorylation of T160 results in increased mobility of Cdk2 during gel electrophoresis and the reaction was monitored by SDS-page. The site of modification was subsequently verified by western blot using an anti-T160 antibody (Cell Signalling 2561) and complete mono-modification was confirmed by intact protein mass spectrometry.

O. Kinase assay

Kinase assays were performed by coupling the conversion of ATP into ADP to the oxidation of NADH (Abs = 340 nm) into NAD^+, mediated by lactate dehydrogenase (LDH) and pyruvate kinase (PK). WT, mutant or ligand-conjugated pCdk2 (12.5 or 50 nM) was preincubated with Cyclin A2 (20 nM) in a buffer containing 50 mM Tris/HCl, 10 mM $MgCl_2$, 5 mM DTT, 1 mM phosphoenolpyruvate, 0.24 mM NADH, 10 U mL^{-1} LDH, 14 U mL^{-1} PK and 5% DMSO at 37 ˚C for 30 minutes. Reactions were initiated by addition of 0.5 mM ATP and 0.24 mM substrate peptide (PKTPKKAKKL) and the absorbance at 340 nm was monitored for two hours. Assays were conducted at 37˚C and carried out in clear 384-well plates (Nunc) using a SpectraMax i3x plate reader (Molecular Devices) with a final volume of 20 µL in each well. After plotting Abs_{340} against time, enzyme velocity was measured from the gradient during the linear phase of product accumulation.

P. TdCD

Circular dichroism was run on a Chirascan spectrometer (Applied Photophysics). A 0.1 cm path-length cuvette was loaded with 300 µL of 10 µM WT, mutant or modified Cdk2 (10 mM HEPES, 100 mM NaCl, 1 mM DTT, pH 7.5). For TdCD measurements, the wavelength was adjusted to 230 nm and elipticity was measured at 1˚C intervals from 25˚C to 80˚C, with for 10 repeats of 10 s integrations per temperature. The melt-temperature (T_m) was determined using Prism 6.0 by fitting a sigmoidal trace to the data, with the quoted T_m being the average of triplicate measurements.

Q. *In cell* target engagement assay

HeLa cells were grown in 35 mm plates to ~95% confluence, before incubating with acrylamide **10** (100, 20 or 4 µM) in 2 mL of media (final DMSO concentration = 0.5%) at 37 °C for 6 hours. Cells were then washed with PBS buffer (3 × 3 mL) and lysed for 20 minutes in RIPA lysis buffer (150 µL, pH 7.5) containing protease inhibitors phenylmethylsulfonyl fluoride (1 mM) and benzamidine.HCl (1 mM). The resulting mixture was centrifuged (13,000 rpm, 20 min, 4˚C) and the supernatant collected. The cell lysates were diluted to 1 mg mL^{-1} in RIPA lysis buffer, as quantified by the DC protein assay (Bio-Rad). Aliquots of 100 µL were precipitated using chloroform/MeOH and resuspended in 25 µL PBS containing 1% SDS. Click chemistry was

performed by the addition of 100 µM TAMRA-azide-biotin[21] tag, 1 mM TCEP, 100 µM TBTA and 1 mM $CuSO_4$. Samples were allowed to react at room temperature for 1 hour and then the proteins were precipitated using chloroform/MeOH and resuspended in 100 µL PBS containing 1% SDS. An aliquot of 5 µL was added to 50 µL 2X SDS-loading buffer and set-aside for western blot analysis. The remaining 95 µL were incubated overnight with 50 µL Neutravadin-agarose beads in PBS at a final volume of 400 µL containing 0.25% SDS. After washing the beads (×5) with 400 µL PBS containing 2% SDS, proteins were eluted by boiling the beads in 50 µL of 5X SDS loading buffer for 5 minutes. For western blot analysis, 12.5 µL of lysate and pulled-down proteins were separated by SDS-page on a 12.5% acrylamide gel, before transferring onto a nitro-cellulose membrane for subsequent primary and secondary antibody incubation. Western blots were visualized by chemiluminescence using HRP-conjugated secondary antibodies.

R. Immunoblot analysis

For immunoblotting, proteins were transferred to nitrocellulose membranes, membranes were blocked (5 % dried skimmed milk in TBS containing 0.1 % Tween-20), then incubated with the appropriate primary, then secondary antibodies in blocking solution, and developed with Luminata Crescendo Western HRP substrate (Millipore) according to the manufacturer's instructions using a Fujifilm LAS 3000 imager. Primary antibodies: anti-Cdk2 (Santa Cruz sc163) and anti-Cdk1 (Santa Cruz sc54), were used at 1:1000 in blocking solution. Secondary antibodies: anti-rabbit and anti-mouse IgG-HRP secondaries (Jackson Labs) were used at 1:10000.

Supplementary Reference

[1] Q. Liu, F.-P. Zhu, X.-L. Jin, X.-J. Wang, H. Chen, L.-Z. Wu, *Chem. - A Eur. J.* **2015**, *21*, 10326–10329.

[2] C. E. Allen, P. R. Curran, A. S. Brearley, V. Boissel, L. Sviridenko, N. J. Press, J. P. Stonehouse, A. Armstrong, *Org. Lett.* **2015**, *17*, 458–460.

[3] G. W. Bemis, M. A. Murcko, *J. Med. Chem.* **1996**, *39*, 2887–2893.

[4] T. Sander, J. Freyss, M. von Korff, C. Rufener, *J. Chem. Inf. Model.* **2015**, *55*, 460–473.

[5] M. Congreve, R. Carr, C. Murray, H. Jhoti, *Drug Discov. Today* **2003**, *8*, 876–877.

[6] H. Köster, T. Craan, S. Brass, C. Herhaus, M. Zentgraf, L. Neumann, A. Heine, G. Klebe, *J. Med. Chem.* **2011**, *54*, 7784–7796.

[7] F. W. Studier, *Protein Expr. Purif.* **2005**, *41*, 207–234.

[8] M. J. Abraham, T. Murtola, R. Schulz, S. Páll, J. C. Smith, B. Hess, E. Lindahl, *SoftwareX* **2015**, *1–2*, 19–25.

[9] A. K. Malde, L. Zuo, M. Breeze, M. Stroet, D. Poger, P. C. Nair, C. Oostenbrink, A. E. Mark, *J. Chem. Theory Comput.* **2011**, *7*, 4026–4037.

[10] A. Fiser, A. Sali, *Bioinformatics* **2003**, *19*, 2500–2501.

[11] R. T. McGibbon, K. A. Beauchamp, M. P. Harrigan, C. Klein, J. M. Swails, C. X. Hernández, C. R. Schwantes, L.-P. Wang, T. J. Lane, V. S. Pande, *Biophys. J.* **2015**, *109*, 1528–1532.

[12] W. Kabsch, *Acta Crystallogr. Sect. D Biol. Crystallogr.* **2010**, *66*, 125–132.

[13] G. Winter, *J. Appl. Crystallogr.* **2010**, *43*, 186–190.

[14] A. J. McCoy, R. W. Grosse-Kunstleve, P. D. Adams, M. D. Winn, L. C. Storoni, R. J. Read, *J. Appl. Crystallogr.* **2007**, *40*, 658–674.

[15] A. A. Lebedev, A. A. Vagin, G. N. Murshudov, *Acta Crystallogr. Sect. D Biol. Crystallogr.* **2008**, *64*, 33–39.

[16] P. V. Afonine, R. W. Grosse-Kunstleve, N. Echols, J. J. Headd, N. W. Moriarty, M. Mustyakimov, T. C. Terwilliger, A. Urzhumtsev, P. H. Zwart, P. D. Adams, *Acta Crystallogr. Sect. D Biol. Crystallogr.* **2012**, *68*, 352–367.

[17] P. Emsley, B. Lohkamp, W. G. Scott, K. Cowtan, *Acta Crystallogr. Sect. D Biol. Crystallogr.* **2010**, *66*, 486–501.

[18] A. A. Lebedev, P. Young, M. N. Isupov, O. V Moroz, A. A. Vagin, G. N. Murshudov, *Acta Crystallogr. Sect. D Biol. Crystallogr.* **2012**, *68*, 431–440.

[19] G. N. Murshudov, A. A. Vagin, E. J. Dodson, *Acta Crystallogr. Sect. D Biol. Crystallogr.* **1997**, *53*, 240–255.

[20] G. N. Murshudov, A. A. Vagin, A. Lebedev, K. S. Wilson, E. J. Dodson, *Acta Crystallogr. Sect. D Biol. Crystallogr.* **1999**, *55*, 247–255.

[21] M. H. Wright, B. Clough, M. D. Rackham, K. Rangachari, J. A. Brannigan, M. Grainger, D. K. Moss, A. R. Bottrill, W. P. Heal, M. Broncel, et al., *Nat. Chem.* **2013**, *6*, 112–121.
